# A UVR-sensor wearable device intervention to reduce sun exposure in melanoma survivors: Results from a randomized controlled trial

**DOI:** 10.1371/journal.pone.0281480

**Published:** 2023-02-10

**Authors:** Rachel Isaksson Vogel, Xianghua Luo, Katherine Brown, Patricia Jewett, Allison C. Dona, Rebekah H. Nagler, Rehana L. Ahmed, Brian C. Martinson, DeAnn Lazovich

**Affiliations:** 1 Department of Obstetrics, Gynecology and Women’s Health, Division of Gynecologic Oncology, University of Minnesota, Minneapolis, Minnesota, United States of America; 2 Masonic Cancer Center, University of Minnesota, Minneapolis, Minnesota, United States of America; 3 Division of Biostatistics, School of Public Health, University of Minnesota, Minneapolis, Minnesota, United States of America; 4 Department of Medicine, Division of Hematology and Oncology, University of Minnesota, Minneapolis, Minnesota, United States of America; 5 Medical School, University of Minnesota, Minneapolis, Minnesota, United States of America; 6 Hubbard School of Journalism & Mass Communication, University of Minnesota, Minneapolis, Minnesota, United States of America; 7 Department of Dermatology, University of Minnesota, Minneapolis, Minnesota, United States of America; 8 HealthPartners Institute, Bloomington, Minnesota, United States of America; 9 Center for Care Delivery and Outcomes Research, Minneapolis Veterans Affairs Health System, Minneapolis, Minnesota, United States of America; 10 Department of Medicine, University of Minnesota, Minneapolis, Minnesota, United States of America; 11 Division of Epidemiology and Community Health, School of Public Health, University of Minnesota, Minneapolis, Minnesota, United States of America; IPATIMUP/i3S, PORTUGAL

## Abstract

**Background:**

Melanoma survivors are at increased risk of developing a second primary melanoma; however, some report sub-optimal sun behaviors and sunburns. We tested the effectiveness of a wearable device with ultraviolet radiation (UVR)-sensing technology to improve sun behaviors and reduce sunburns in cutaneous melanoma survivors.

**Materials and methods:**

We conducted a randomized controlled trial using Shade 2, a commercially available wrist device that measures UVR. The intervention group received the device and mobile application notifications about their exposure and prompts to use sunscreen. The control group received the device and a separate research mobile application without information about their exposure or notifications. Participants wore the device for 12 weeks and self-reported sun behaviors before, during, and after the intervention. The primary outcome was a composite score of sun protection behaviors at week 12.

**Results:**

386 participants were randomized (186 control, 182 intervention). Most were female and 5+ years past their first melanoma diagnosis. The average age was 56 years. Most (93%) completed the study, though 40% experienced device issues. No meaningful differences were observed in self-reported sun protection behaviors at week 12 (controls 3.0±0.5 vs. intervention 2.9±0.5, p = 0.06), any sunburn during the intervention period (controls 14.4% vs. intervention 12.7%, p = 0.75), or average daily objective UVR exposure (controls median 87 vs. intervention 83 J/m^2^, p = 0.43).

**Conclusion:**

Wearing a device that measured and alerted melanoma survivors to UVR exposure did not result in different sun behaviors, exposure, or sunburns relative to controls. The technology needs refinement before further attempts to assess the effectiveness of self-monitoring UVR exposure.

**Clinical trials registration:**

NCT03927742.

## Introduction

Approximately 100,000 people in the United States (US) will be diagnosed with melanoma in 2022, and by 2030, there will be a projected 1.8 million melanoma survivors [1]. Melanoma survivors are at significantly greater risk of developing a second primary melanoma [2]. Ultraviolet radiation (UVR) exposure is the strongest risk factors for melanoma [3–5]. While melanoma survivors generally report less UVR exposure and more protective sun behaviors [6]—such as utilizing shade, protective clothing, and sunscreen—than those without melanoma, a nontrivial number of survivors report suboptimal sun exposure [7, 8]. Therefore, interventions to promote healthy sun behaviors among melanoma survivors are needed to prevent subsequent melanomas in this population.

Few interventions have targeted sun protective behaviors in melanoma survivors specifically, despite their high risk for future diagnoses. Most behavioral interventions to date have been designed for those without a previous melanoma diagnosis, most often school-aged children or young adults, high-risk occupational groups, or individuals otherwise at elevated risk for melanoma, such as family members or solid-organ transplant recipients (e.g. [9, 10]). The few prior melanoma survivor interventions have primarily focused on skin self-examinations [11–16]. Two studies, both web-based, also evaluated sun behaviors in melanoma survivors, with moderate improvements for some behaviors at various time points [11, 14].

Many preventive health interventions have drawn from the Health Belief Model, emphasizing risk perceptions and cues to action for promoting behavior change [17, 18]. Technology can be useful to implement these drivers [19]. Wearable technologies, such as smart watches, have become widely used and provide a novel means for health intervention. Some new wearable technologies incorporate UVR sensors to track exposure and prompts to reduce exposure, which may be especially important to reduce unintentional sun exposure.

The objective of this trial was to test the effectiveness of a wearable device with UVR-sensing technology coupled with a mobile application to promote healthy sun behaviors in melanoma survivors. We hypothesized that participants randomized to the intervention, compared to the control participants, would report greater sun protection and avoidance practices and fewer sunburns following a 12-week intervention.

## Materials and methods

### Overview and study design

Details regarding the protocol including the study design and schematic, intervention and comparator choice, eligibility and recruitment methods, randomization procedures, measures, and sample size considerations are described elsewhere [19]. Briefly, we conducted the MELD (Melanoma Exposures and Life after Diagnosis) study, a randomized controlled trial among 368 melanoma survivors over two waves (Summer 2020 and 2021). This two-wave summer method was utilized to account for seasonality of UVR exposure in the Midwest and allow for adequate sample size recruitment. Participants were randomly assigned 1:1 to the intervention or control group for 12 weeks and surveyed regarding their sun protection and surveillance behaviors at study entry and every 4 weeks during the intervention period. All participants provided informed consent prior to study enrollment. The study received approval from the University of Minnesota Institutional Review Board (STUDY00006257) and HealthPartners Institute Institutional Review Board (A18-353), and all participants provided written informed consent. The trial was registered on ClinicalTrials.gov (NCT03927742).

### Recruitment

Individuals diagnosed with or treated for invasive cutaneous melanoma between 2010 and 2020 were recruited from HealthPartners healthcare system, a large consumer-governed nonprofit healthcare organization based in Minnesota, from May through July 2020 and 2021. Eligible participants were 18–75 years old, previously diagnosed with stage I–IV cutaneous invasive melanoma, able to read/write in English, owners of a smartphone, and able to provide voluntary informed consent.

Potential eligible individuals were identified via electronic health records and were mailed a letter introducing the study and elements of informed consent. Within 2 weeks of sending the letter, potential participants were contacted via phone to determine interest in participating in the study. Interested individuals were sent an introductory email from the University of Minnesota team including a link to online consent and Health Insurance Portability and Accountability Act (HIPAA) forms, which then led directly to the online baseline survey in Research Electronic Data Capture (REDCap) [20]. Potential participants were encouraged to reach out to the study team with questions or concerns prior to and after providing consent.

### Randomization

Following written consent and completion of the baseline survey, participants were randomized 1:1 to the intervention or control arm. Randomization was stratified by age (<50, 50+ years old), gender (male, female), disease stage (I, II, III/IV, unknown), and years since first melanoma diagnosis (< 2 years, 2–5 years, 6+ years). Randomization was implemented within REDCap by the study coordinator. Everyone except the study coordinator and statistician were blinded to the randomization. Participants were not informed of their randomized group or provided specific information about what to expect from the mobile app.

### Intervention

Participants were provided a commercially available UVR-sensor-enabled wrist wearable device and asked to wear the device and sync it with the mobile app every day for 12 weeks regardless of planned outdoor activities, except while swimming or doing water sports (device is not waterproof). We originally planned to use the Microsoft Band 2 that measured UVR exposure and additional health behaviors, such as steps and heart rate; it also sent notifications about using sunscreen, wearing a hat, seeking shade, and minimizing midday sun exposure. The Microsoft device, however, was discontinued and no longer supported by the time of study initiation. Instead, we used the Shade 2^nd^ generation device, the only other commercially available UVR-sensor-enabled wrist wearable at the time. Shade was significantly more accurate in measuring sun exposure than similar devices, including the Microsoft Band [21]. However, the Shade device does not collect information on other health behaviors beyond UVR exposure, and messaging was not customizable and focuses solely on reminders to apply sunscreen.

While all participants were provided the same device, they used different versions of an associated mobile app based on their randomization. Intervention participants received the commercially available version of the mobile app, which helped users set daily UVR exposure limits, taking into account UVR intensity, time of exposure, and sunscreen application. When approaching their daily UVR limit, smartphone notifications were sent to users as a cue to action. The mobile app version used by participants in the control group only collected UVR exposure, not sunscreen use, and did not provide notifications or information about measured UVR exposure to participants. They only received a message indicating that their UVR exposure data had been reported to the study team. We considered multiple options at the time of study design and ultimately chose the attention-control group for three reasons: one, we can objectively measure UVR exposure in both groups; two, providing the device to the control group will help with recruitment, engagement, and retention; and three, the intervention is low risk.

Participants in the intervention group also received a study brochure with information about the importance of UVR avoidance and protection, as well as tips for making sun-safe behaviors routine and sustainable. This content was provided both as a printed brochure at baseline and via a website, viewable after completing each subsequent survey. Participants in the control group did not receive the brochure nor were they directed to the website.

### Data collection and measures

Due to the COVID-19 pandemic, all study procedures were conducted remotely. Study measures were collected via online surveys and the Shade device. Participants were mailed their device, training materials, and mobile app access at the time of randomization. Participants were asked to wear the device and sync it with the mobile app every day for 12 weeks. The study coordinator monitored device use of all participants and contacted participants who did not use their device or sync the app data on more than 3 days in any given week to determine barriers and encourage use. We used a weekly $100 raffle-drawing incentive to promote adherence. Almost half (40%) of participants unexpectedly experienced broken straps or device malfunction during the study. Damaged or defective devices were promptly replaced; nevertheless, many participants were without a functioning device for approximately 5–10 days during the study.

Self-administered questionnaire data were collected at baseline, week 4, week 8, and week 12 (end of intervention), as well as week 64 (end of the summer, one year after intervention completion) to assess potential continued behavior change.

The primary outcome was a composite measure of sun protection behaviors (sun habit score) at the end of the intervention period (12-week survey) [22]. A total sun habit score for each individual was created by taking the average of 6 protective behaviors (wearing a shirt with sleeves, wearing sunglasses, staying in the shade, using sunscreen, spending time in the sun to get a tan [reverse coded], and wearing a hat) measured on a 5-point ordinal scale ranging from 1 (never) to 5 (always), recoded to 1 (never or rarely) to 4 (always) to align with previously published studies.

Secondary outcomes included a) sunburns (the proportions of individuals who self-reported at least one red or painful sunburn in the past 3 months at the 12-week survey) [23] and b) UVR exposure, which was both self-reported (number of hours spent outside per day between 10am and 4pm in the summer separately for weekdays and weekend days) and automatically measured by the Shade device (instantaneous UVR intensity and accumulated UVR dose over time). The device collects exposure over the course of the day and reports accumulated joules per meter squared (J/m^2^) every 6 minutes. If a participant in the intervention group entered into the mobile app that they applied sunscreen, the Shade commercial mobile app mathematically reduced the cumulative UVR exposure that was reported to the participant for the duration of 90 minutes. Therefore, for the following 90 minutes, these individuals did not receive reports about the true incoming UVR, but a modified (reduced) UVR exposure calculated as the objective UVR exposure divided by the SPF of the reported sunscreen. Furthermore, these modified data were provided by Shade as the raw data through the research portal for participants in the intervention group. For our analyses, we back-transformed this modified exposure estimate if individuals had UVR exposure and reported sunscreen use, using the time-stamped sunscreen application and SPF data. In this manuscript, we refer to unmodified objective UVR exposure (among those who did not report using sunscreen) and to the back-transformed UVR exposure (among those who used sunscreen at the time) as ‘objective UVR exposure’. We refer to the UVR exposure that was reduced by the SPF factor as ‘SPF modified UVR exposure’. All UVR data in the control group were objective UVR exposures.

We measured self-reported physical activity, depression, and anxiety to examine potential unintended negative consequences of the intervention. Physical activity was measured using the Godin Leisure-Time Exercise Questionnaire [24], which asks about times and minutes per week of engaging in strenuous (vigorous), moderate, and mild physical activity on average during the preceding month. Depression and anxiety were measured using the 14-item Hospital Anxiety and Depression Scale (HADS) instrument [6]. We measured satisfaction and usability of the device at week 12 using items from the System Usability Scale [25].

### Statistical considerations

We calculated the sample size to achieve high power for the primary outcome (sun habit score) while also maintaining moderate power for experience of a sunburn during the intervention period, a key secondary outcome. A sample size of 314 randomized achieves 95% power to detect an effect size of 0.4 between intervention and control groups using a two-sided two-sample t-test assuming a significance level of 0.05. This sample size also achieves 70% power to detect 20% of the control participants reporting a sunburn and 10% of the intervention participants reporting a sunburn as statistically significant at a significance level of 0.05. We estimated that 85% of those randomized would complete the 12-week follow-up survey, resulting in a final calculated sample size of 368.

Participant demographic characteristics and sun behaviors reported at baseline were summarized using descriptive statistics. The main analysis of the primary outcome, total sun habit scores, was analyzed using two-sided two-sample t-test comparing scores at the end of the intervention period (12 weeks) between intervention and control groups. A secondary analysis explored changes in the total sun habit scores over the intervention period (baseline, weeks 4, 8 and 12) using a linear mixed effects regression model with fixed effects of time, group and time-group interactions, assuming that data were missing at random (MAR) [26]. We further analyzed the data at week 64. Because the groups were balanced across demographic characteristics, adjustment was unnecessary. Pre-planned subgroup analyses for gender (female, male), age (<50, 50+ years old), time since first diagnosis (0–5 years, 5+ years), stage (I, II/III, IV), and study wave (2020, 2021) were conducted for the primary outcome at week 12 by fitting linear regression models with the treatment indicator, subgroup indicator(s), and their interactions. All analyses were conducted following intention-to-treat procedures: all subjects were followed up, evaluated, and analyzed as members of their randomized group regardless of their compliance with the assigned intervention.

Comparisons of secondary outcomes—including experience of sunburn, self-report sun behaviors, skin cancer knowledge, emotional health, and physical activity—by randomization group were conducted using Chi-squared and t-tests as appropriate. Individual objective UVR data were summarized as minutes of UVR exposure per day, exposure per weekday, exposure per weekend day, and per day between 10am and 4pm (peak hours). Because the UVR data were skewed to the right with a few large exposures, median exposure levels were calculated and compared by randomization group using Wilcoxon rank sum tests and within the intervention group using Wilcoxon signed rank test. Descriptive statistics were used to summarize satisfaction and usability. Surveys had minimal missing data, and with little loss-to-follow-up, we analyzed the complete data only. All tests were two-sided. Data were analyzed using SAS 9.4 and p-values <0.05 were considered statistically significant.

## Results

**Fig 1:**
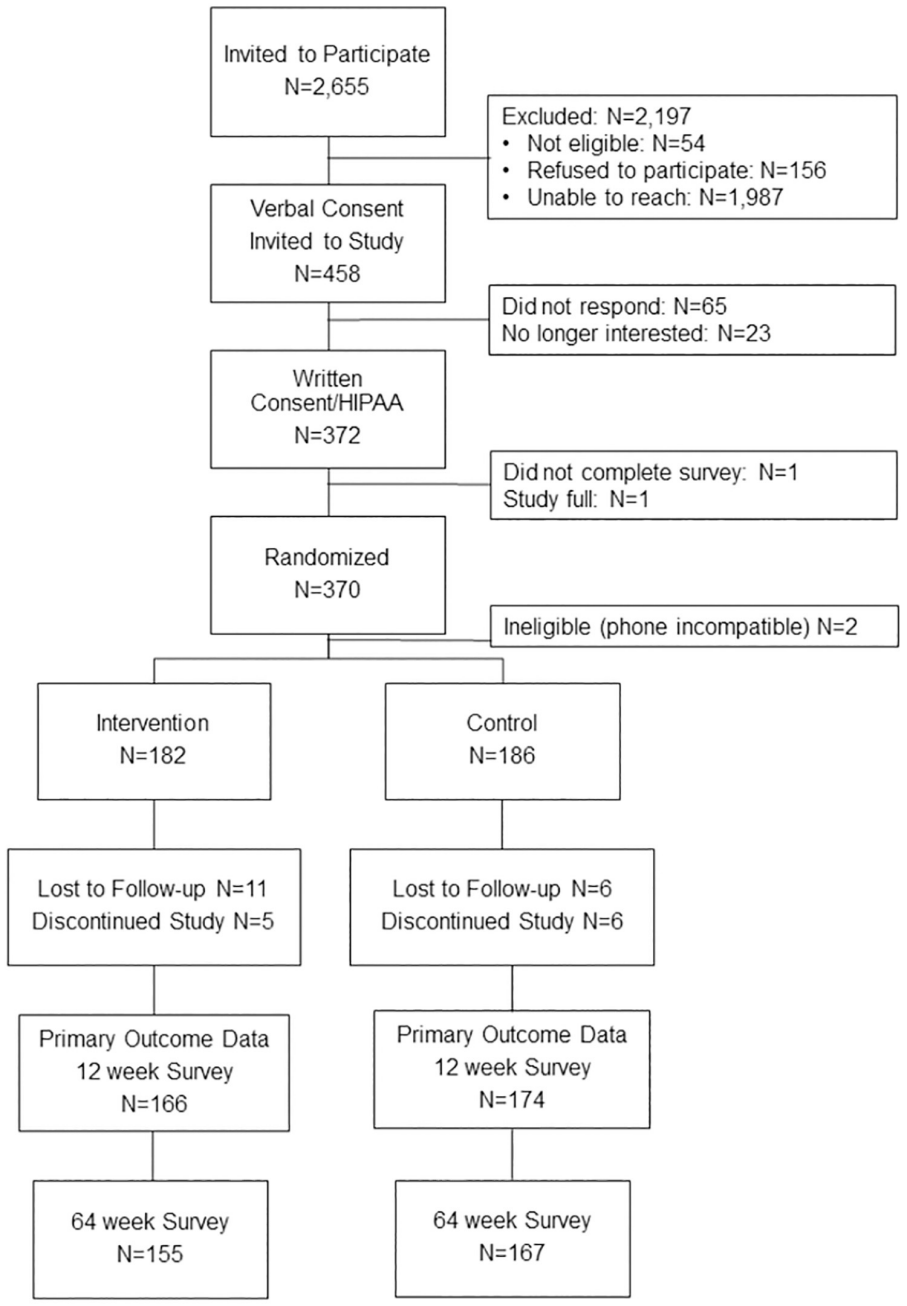
CONSORT diagram.

A total of 2,655 individuals were invited to participate; 458 provided verbal consent for their names and email to be passed to the University of Minnesota team, 372 provided written consent, and 370 completed the baseline survey and were randomized (Fig 1). Following randomization, it was determined that two participants did not have a phone compatible with the mobile app and were therefore ineligible, resulting in the planned 368 participants, 182 randomized to intervention and 186 randomized to control. In wave 1 (May-July 2020), 25% of participants were recruited and randomized (n = 92), with the remainder recruited in wave 2 (May-July 2021).

Participants were on average 56 years old (24–75 years), and most (60%) were female, highly educated, and married or partnered (Table 1). Most reported stage I disease, though approximately 1/3 were not aware of their stage. More than half were >5 years from their first melanoma diagnosis. Approximately 20% reported at least one additional (or subsequent) primary melanoma diagnosis.

**Table 1:** Baseline demographics of the intervention and control participants (N = 368).

	**Control N = 186**		**Intervention N = 182**	
	**N**	**Mean (SD)**	**N**	**Mean (SD)**
**Age, years**	186	55.3 (11.5)	182	56.5 (11.9)
**Body mass index, kg/m** ^**2**^	181	28.0 (5.1)	179	28.0 (5.7)
	**N**	**%**	**N**	**%**
**Gender**				
Female	110	59.1	111	61.0
Male	76	40.9	71	39.0
**Race**				
White, Non-Hispanic	171	91.9	174	95.6
Other	15	8.1	8	4.4
**Education**				
High school graduate or less	14	7.6	12	6.6
Vocational/business school or AA degree	28	15.1	39	21.6
Some college	23	12.4	17	9.4
College graduate	64	34.6	70	38.7
Graduate or professional training	56	30.3	43	23.8
**Income**				
<$20,000	1	0.5	0	0.0
$20,000-$49,999	11	5.9	13	7.2
$50,000-$74,999	23	12.4	21	11.6
$75,000-$99,999	35	18.9	36	19.9
$100,000-$149,999	46	24.9	43	23.8
$150,000-$199,999	21	11.4	31	17.1
$200,000 or more	25	13.5	24	13.3
Prefer not to say	23	12.4	13	7.2
**Marital Status**				
Never married	13	7.0	15	8.2
Married / partnered	160	86.0	147	80.8
Widowed	0	0.0	4	2.2
Divorced	13	7.0	16	8.8
**Time since first melanoma diagnosis**				
0–1.99 years	21	11.3	19	10.4
2–5 years	52	28.0	56	30.8
>5 years	113	60.8	107	58.8
**Melanoma Location**				
Head or neck	24	12.9	34	18.7
Trunk (stomach or back)	52	28.0	44	24.2
Arms (including shoulder)	48	25.8	35	19.2
Legs	46	24.7	53	29.1
Other	16	8.6	16	8.8
**Stage**				
I	82	44.1	81	44.5
II	20	10.8	20	11.0
III	17	9.1	16	8.8
IV	7	3.8	5	2.8
Unknown	60	32.3	60	33.0
**Secondary primary**				
No	151	81.2	137	75.7
Yes	35	18.8	44	24.3

Study retention was high, with 92.4% of participants (91.2% intervention, 93.5% control) completing the 12-week survey and 87.5% of participants (85.2% intervention, 89.8% controls) completing the 64-week survey. Participants at baseline reported overall high sun protection scores, with average protection scores near 3.0 indicating that participants “often” engaged in most of the protection behaviors. At 12 weeks, no statistically or clinically meaningful differences were found between groups for the primary outcome, the sun habit score (control 3.0±0.5 vs. intervention 2.9±0.5, t(337) = 1.90, p = 0.06; effect size = 0.21). Scores remained stable over the intervention period, both overall and for each separate behavior, and the repeated measures analysis showed no significant difference in overall or individual scores between the two arms during the intervention period (Table 2). At 64 weeks, the results remained similar; no differences were observed between groups for the sun habit score (control 3.2±0.5 vs. intervention 3.2±0.5, t(311) = 0.42, p = 0.67; effect size = 0.05).

**Table 2:** Differences in least square (LS) means for sun protection scores at baseline and during the intervention period between the intervention and control groups.

	Baseline		4 weeks		8 weeks		12 weeks		p-value^1^
	Mean difference^2^	95% CI	Mean difference^2^	95% CI	Mean difference^2^	95% CI	Mean difference^2^	95% CI	
**Sun habit score** ^3^	-0.09	(-0.18, 0.01)	-0.06	(-0.15, 0.04)	-0.07	(-0.17, 0.03)	-0.10	(-0.20, 0.00)	0.71
Wear sunscreen^4^	-0.17	(-0.40, 0.05)	-0.28	(-0.51, -0.05)	-0.31	(-0.54, -0.08)	-0.27	(-0.50, -0.04)	0.57
Shirt with sleeves^4^	-0.04	(-0.26, 0.16)	0.04	(-0.17, 0.26)	0.09	(-0.13, 0.31)	-0.09	(-0.31, 0.12)	0.27
Wide brim hat^4^	-0.12	(-0.39, 0.15)	-0.08	(-0.36, 0.19)	-0.13	(-0.40, 0.15)	-0.02	(-0.30, 0.25)	0.70
Stay in shade^4^	-0.11	(-0.30, 0.07)	-0.04	(-0.22, 0.15)	-0.01	(-0.19, 0.18)	-0.06	(-0.25, 0.13)	0.67
Wear sunglasses^4^	-0.16	(-0.39, 0.07)	-0.08	(-0.31, 0.15)	-0.17	(-0.40, 0.06)	-0.25	(-0.48, -0.01)	0.14
Spend time in sun to get tan^5^	0.04	(-0.09, 0.16)	0.04	(-0.09, 0.17)	-0.03	(-0.16, 0.09)	0.02	(-0.10, 0.15)	0.62

^1^ Test for any difference between the two arms at week 4, 8 or 12 in terms of their changes from baseline (H_0_: the interaction effects between time and group = 0 based on the linear mixed model with fixed effects of time [reference = baseline], group [reference = control], and time-group interactions).

^2^ Least square mean difference; intervention minus control

^3^ Mean score of six measures: wear sunscreen, shirt with sleeves, hat with wide brim, stay in shade, wear sunglasses, in sun to get tan (reverse coded); higher is better sun protection behaviors

^4^ 1 = Never/rarely, 4 = Always

^5^ 1 = Always, 4 = Never/rarely

Pre-planned subgroup analyses (test for interaction effects) did not reveal differences in the primary outcome (Table 3). We also stratified by baseline self-reported weekday and weekend sun exposure and did not observe differences by subgroup.

**Table 3:** Differences in sun protection scores at week 12 between the intervention and control groups across subgroups.

	Week 12 sun habit score					p-value^2^
	Control		Intervention		Difference^1^	
Subgroups	N	Mean (SD)	N	Mean (SD)	Mean (95% CI)	
**Gender**						0.21
Female	104	2.9 (0.5)	105	2.9 (0.5)	-0.05 (-0.18, 0.09)	
Male	70	3.1 (0.4)	60	2.9 (0.5)	-0.18 (-0.35, -0.02)	
**Age**						0.74
<50 years	51	2.9 (0.4)	44	2.8 (0.6)	-0.08 (-0.28, 0.13)	
≥50 years	123	3.0 (0.5)	121	2.9 (0.5)	-0.11 (-0.24, 0.01)	
**Time since diagnosis**						0.79
0–5 years	100	2.9 (0.4)	101	2.8 (0.5)	-0.11 (-0.24, 0.02)	
>5 years	74	3.0 (0.5)	64	3.0 (0.5)	-0.08 (-0.25, 0.09)	
**Stage**						0.22
I	77	3.1 (0.4)	74	2.9 (0.5)	-0.18 (-0.33, -0.04)	
II/III	33	3.0 (0.4)	32	2.8 (0.7)	-0.18 (-0.46, 0.10)	
IV	7	3.0 (0.6)	4	3.2 (0.5)	0.14 (-0.66, 0.94)	
Unknown	57	2.8 (0.5)	55	2.9 (0.5)	0.04 (-0.14, 0.22)	
**Study Wave**						0.26
2020	40	3.0 (0.5)	42	2.8 (0.5)	-0.21 (-0.42, 0.01)	
2021	134	3.0 (0.5)	123	2.9 (0.5)	-0.06 (-0.18, 0.05)	
**Baseline weekday sun exposure** ^3^						0.92
1 hour or less	100	3.0 (0.5)	109	2.9 (0.5)	-0.10 (-0.24, 0.04)	
2 hours+	74	3.0 (0.4)	56	2.9 (0.5)	-0.09 (-0.24, 0.07)	
**Baseline weekend sun exposure** ^3^						0.67
1 hour or less	27	3.1 (0.4)	33	3.0 (0.5)	-0.16 (-0.40, 0.09)	
2 hours+	147	3.0 (0.5)	132	2.9 (0.5)	-0.10 (-0.21, 0.02)	

^1^Mean difference, intervention minus control

^2^Type 3 test p-value for the interaction based on the linear regression with the treatment indicator, subgroup indicator(s), and their interaction(s)

^3^During peak hours, 10am-4pm

At baseline, both groups reported moderate sun exposure, with 80% reporting more than 2 hours of sun exposure on average during peak hours on the weekend and approximately 30% reporting a sunburn in the previous year. During the intervention period (assessed at week 12), 25 (14.4%) participants in the control group and 21 (12.7%) in the intervention group reported at least one sunburn (p = 0.75; Table 4). No statistically significant differences in self-reported weekday or weekend sun exposure during peak hours were observed at any time point, with the exception of fewer participants in the intervention group reporting being outside for 2 hours or more at week 64 on weekend than those in the control group (64.0% vs. 74.5%, p = 0.04).

**Table 4:** Sunburns and participant reported sun exposure at baseline and during the intervention period by MELD randomization group.

	Baseline^1^		4 weeks^2^			8 weeks^2^			12 weeks^3^			64 weeks^1^		
	Control N = 186	Int N = 182	Control N = 175	Int N = 162		Control N = 171	Int N = 162		Control N = 174	Int N = 165		Control N = 167	Int N = 155	
Secondary Outcomes	N (%)	N (%)	N (%)	N (%)	p-value	N (%)	N (%)	p-value	N (%)	N (%)	p-value	N (%)	N (%)	p-value
**Red/painful sunburn one day or more**					0.66			0.34			0.75			0.06
None	132 (71.0)	126 (69.6)	163 (93.1)	153 (94.4)		164 (95.9)	159 (98.1)		149 (85.6)	144 (87.3)		144 (87.3)	119 (79.3)	
1 or more	54 (29.0)	55 (30.4)	12 (6.9)	9 (5.6)		7 (4.1)	3 (1.9)		25 (14.4)	21 (12.7)		21 (12.7)	31 (20.7)	
**Weekday sun exposure** ^4^					0.35			0.07			0.30			0.10
1 hour or less	107 (57.5)	122 (67.0)	97 (55.4)	98 (60.5)		91 (53.2)	102 (63.0)		111 (63.8)	114 (69.1)		107 (64.8)	110 (73.3)	
2 hours+	79 (42.5)	60 (33.0)	78 (44.6)	64 (39.5)		80 (46.8)	60 (37.0)		63 (36.2)	51 (30.9)		58 (35.2)	40 (26.7)	
**Weekend sun exposure** ^4^					0.71			0.51			0.17			0.04
1 hour or less	31 (16.7)	37 (20.3)	36 (20.6)	36 (22.2)		40 (23.4)	43 (26.5)		46 (26.4)	55 (33.3)		42 (25.5)	54 (36.0)	
2 hours+	155 (83.3)	145 (79.7)	139 (79.4)	126 (77.8)		131 (76.6)	119 (73.5)		128 (73.6)	110 (66.7)		123 (74.5)	96 (64.0)	

^1^ Previous summer

^2^ Previous 4 weeks

^3^ Previous 12 weeks

^4^ During peak hours, 10am-4pm

Int: Intervention

Most participants downloaded the mobile app, used the device, and provided at least some automatically collected UVR exposure data (92.1% overall; 90.0% intervention and 94.1% controls). Crossover was minimal; four control participants downloaded and used the commercially available (intervention) mobile application; no one in the intervention group downloaded the control mobile app. The median number of days with objective UVR data was 59 days (range: 0–126) for the intervention group and 53 days (range: 0–97; p = 0.06) for the control group. Approximately 75% of participants reported wearing the device all of the time at least 5 days a week, and this was similar between study arms. The total objective UVR dose measured by the device over the intervention period was similar between groups, both during peak hours (10am-4pm) and during full days (Table 5). In addition, participants in both groups had a similar median number of minutes with any exposure per day and average objective daily dose.

**Table 5:** Objective UVR exposure data during the intervention period by MELD randomization group.

	Control		Intervention		
Measure	N	Median (Range)	N	Median (Range)	p-value
**Days with UVR exposure data during intervention**	186	53 (0–97)	181	59 (0–126)	0.07
**Total dose during intervention (J/m2)**					
All	175	4,005 (6–128,990)	164	4,585 (1–57,004)	0.19
Peak hours (10am-4pm)	174	2,317 (3–69,512)	164	2,867 (1–21,597)	0.11
**Minutes per day of exposure**					
All	175	156 (17–1,071)	164	158 (37–1,012)	0.67
Peak hours (10am-4pm)	174	100 (6–350)	164	91 (31–354)	0.21
**Average daily dose (J/m2)**					
All	175	87 (0.2–1,633)	164	83 (0.2–950)	0.43
Peak hours (10am-4pm)	174	75 (0.4–927)	164	70 (0.1–424)	0.22
Weekdays	175	73 (0.3–1,482)	164	65 (0.1–1,044)	0.50
Peak hours Weekdays	173	57 (0.2–585)	164	51 (0.08–489)	0.57
Weekends	169	101 (0.2–2,023)	164	96 (0.2–1,099)	0.24
Peak hours Weekends	168	88 (0.3–1,854)	163	83 (0.1–909)	0.15

In a post-hoc exploratory analysis, we examined the objective and SPF modified UVR exposure data among those in the intervention group. Most (75%) participants in the intervention group used the sunscreen feature at least once; those who did not were more likely to have stage 3 or 4 disease (24.4% vs. 8.1%, p = 0.007) but were otherwise similar in demographics. Both those who did and did not use the sunscreen feature reported similar minutes per day of exposure (median 160 vs. 151). Comparisons of the objective UVR exposure, however, found that those who reported using sunscreen within the app had greater objective UVR exposure than those who did not, which remained statistically significant even after adjusting for stage (e.g. average daily dose median 87 vs. 56 J/m2; Table 6). In contrast, the SPF modified UVR exposure dose seen by participants who reported using sunscreen was on average 25–30% less than the objective UVR exposure dose.

**Table 6:** SPF modified and objective UVR exposure data during the intervention period among those in the intervention group by use of the sunscreen feature.

	Used sunscreen feature to report sunscreen use				Did not use sunscreen feature		
	SPF modified UVR exposure		Objective UVR exposure		Objective UVR exposure		
Average daily dose (J/m2)	N	Median (Range)	N	Median (Range)	N	Median (Range)	p-value^1^
All	123	63 (0.3–768)	123	87 (0.4–769)	41	56 (0.2–950)	0.03
Weekdays	123	51 (0.3–656)	123	69 (0.4–712)	41	52 (0.1–1,044)	0.36
Weekends	123	89 (0.2–1,043)	123	118 (0.2–1,099)	41	53 (0.2–732)	0.001

^1^ Comparison of objective UVR exposure data between those who did and did not use the sunscreen feature using median regressions, adjusting for stage (I/II, III/IV, unknown).

Concerning physical activity and emotional health, we identified no differences between the control and intervention groups (e.g. strenuous minutes per week 39.8±20.1 vs. 41.1±34.4, p = 0.70), rates of clinically relevant symptoms of anxiety (9.2% vs. 12.7%, p = 0.54) or depression (1.8% vs. 1.2%, p = 0.92) at 12 weeks, respectively (Table 7).

**Table 7:** Potential unintended consequences—physical activity, depression, and anxiety—at week 12 by intervention group.

	**Control**		**Intervention**		
	**N**	**Mean (SD)**	**N**	**Mean (SD)**	**p-value**
**Physical Activity**					
Strenuous Minutes per week	100	39.8 (20.1)	87	41.4 (34.4)	0.70
Moderate Minutes per week	138	41.4 (20.2)	138	39.7 (25.3)	0.55
Mild Minutes per week	165	41.4 (36.0)	158	38.7 (28.1)	0.47
**Hospital Anxiety and Depression Scale**					
Anxiety subscale	173	5.73 (3.77)	164	5.45 (3.49)	0.49
Depression subscale	173	2.84 (2.45)	164	2.76 (2.62)	0.77
	**N**	**%**	**N**	**%**	**p-value**
**Hospital Anxiety and Depression Scale**					
*Anxiety subscale*					0.54
Normal (0–7)	126	72.8	122	74.4	
Borderline (8–10)	25	14.5	27	16.5	
Clinically relevant symptoms (11–21)	22	12.7	15	9.2	
*Depression subscale*					0.92
Normal (0–7)	166	96.0	156	95.1	
Borderline (8–10)	5	2.9	5	3.1	
Clinically relevant symptoms (11–21)	2	1.2	3	1.8	

About half of participants reported being satisfied or very satisfied with the device (Table 8). Those in the intervention group reported paying greater attention to the device and the mobile application, and more often reported that the device affected their sun behaviors compared to the control group (all p<0.0001). While most agreed the device was easy to use, less than half reported they would like to use the system frequently and would continue using the device, even among those in the intervention group. Those assigned to the intervention group, however, were more likely to recommend this device and mobile app to a friend or relative than those in the control group (57.6% vs. 39.5%, p = 0.004). Participant comments about the device centered on concerns about durability and quality, recommendations to integrate the technology into a device they were already wearing with more features, and the need for a waterproof device.

**Table 8:** Device intervention satisfaction at week 12 by intervention.

	**Control**		**Intervention**		
***0 = Not at all*, *100 = Very much***	**N**	**Mean (SD)**	**N**	**Mean (SD)**	**p-value**
**How much did you pay attention to the Shade device?**	150	33.7 (30.3)	129	51.7 (33.2)	<0.0001
**How much did you pay attention to the app on your mobile phone?**	150	26.4 (28.3)	130	57.4 (29.9)	<0.0001
**How much did the Shade device and mobile app affect your sun behaviors?**	150	21.8 (26.7)	129	53.3 (32.3)	<0.0001
	**N**	**%**	**N**	**%**	**p-value**
**How satisfied were you with the Shade device**					0.49
Not at all satisfied	17	10.1	19	11.7	
Somewhat satisfied	65	38.5	50	30.9	
Satisfied	72	42.6	74	45.7	
Very satisfied	15	8.9	19	11.7	
**I think that I would like to use this system frequently.**					0.007
Strongly agree	10	5.8	28	17.1	
Somewhat agree	56	32.6	54	32.9	
Neutral	58	33.7	38	23.2	
Somewhat disagree	29	16.9	32	19.5	
Strongly disagree	19	11.1	12	7.3	
**I thought the system was easy to use.**					0.42
Strongly agree	72	42.1	79	48.2	
Somewhat agree	68	39.8	50	30.5	
Neutral	14	8.2	14	8.5	
Somewhat disagree	12	7.0	17	10.4	
Strongly disagree	5	2.9	4	2.4	
**Would you continue to use this device and mobile app?**					0.41
No	39	22.7	40	24.2	
Yes	66	38.4	72	43.6	
Unsure	67	39.0	53	32.1	
**Would you recommend this device and mobile app to a friend or relative?**					0.004
No	32	18.6	21	12.7	
Yes	68	39.5	95	57.6	
Unsure	72	41.9	49	29.7	

## Discussion

Contrary to our hypotheses, we found no differences in self-reported sun protection behaviors, UVR exposure, or sunburns following the 12-week intervention, including no behavioral differences in subgroup analyses. We further found no long-term behavioral differences with the one exception of slightly fewer participants in the intervention group reporting spending at least 2 hours outside on weekends at 64 weeks. Below we discuss possible reasons for our overall null findings and lessons learned for future research in this area.

We chose a previously validated summary measure of self-reported sun protection behaviors as the primary outcome for this study to enable comparisons with other similar intervention studies [22]. However, the Shade device only included recommendations for sunscreen use and no other protective measures (wearing protective clothing and/or hat, seeking shade, avoiding peak sun), which may have contributed to a lack of change in the summary measure and in these individual behaviors. In a 2021 prospective cohort study among young adults using a UV-dosimeter and messaging and goal-setting app, reductions in chest and shoulder sunburns and a trend towards fewer scalp sunburns over a 28-day period were observed, and wearing protective clothing was the most common goal reported [27]. It is possible that wearing t-shirts and hats are easily achievable sun protection measures among young cancer-free adults who may be likely to wear little protective clothing otherwise. We were unable to provide messaging about protective clothing or other sun protection behaviors. Additionally, in our study population of melanoma survivors, we recorded high levels of sun protective behaviors in the study population already at baseline (both in the intervention and control arms) with overall little room for sun protection improvements. As a self-reported outcome, social desirability to use sun protection may also have affected reported measures, leading to a ceiling effect.

A key secondary outcome for this study was self-report of at least one red or painful sunburn during the intervention period. The National Institutes of Health has deemed sunburns following an intervention to be a clinically relevant endpoint and suitable outcome for trials aiming to prevent skin cancer [28]. Although sun protection behaviors were high at baseline, 30% of participants reported a sunburn in the previous year at baseline, more than previously estimated in a similar population [7], and indicating a need for behavior change for at least some participants. Post-intervention sunburn rates were lower (<15%), which may indicate potential improvements in both groups, or we may have missed sunburns in late spring before the study.

We chose an attention-control group over no intervention so that we could objectively measure UVR exposure in both groups and increase engagement in the control group over the 12-week intervention. A 2021 study that used the Shade device and control mobile application found that unintentional tanning and time spent outside decreased among children (aged 8–17) who wore the device for a two-week period; however, no changes in UVR exposure or sunburn were observed [29]. Notably, the study also observed that sunscreen use decreased over the study period, with the authors concluding this change could dampen the detection of intervention effects. While it is possible that our control group was affected by the device, sun behavior scores were stable over the intervention period, indicating this was likely not the case. In retrospect, however, we acknowledge that a second control group who did not receive the device would have strengthened the study.

Some of the Shade device features may have contributed to our null findings. Although Shade is highly sensitive and accurate, defects in the hardware resulted in many participants needing device repair or replacement during the study. Further, the Shade built-in algorithm, which reported SPF-reduced UVR exposure to participants after sunscreen use, is potentially problematic and misleading, as evidenced by our exploratory finding that those who reported using sunscreen compared with those who did not had higher objective UVR exposure. The goal of using sunscreen is to reduce sunburn, but can mislead sunscreen users to believe they can prolong their direct sun exposure without additional protective measures. Because of this and the general lack of proper use of sunscreen (amount and reapplication), the World Health Organization recommends that other protection methods such as clothing and shade be prioritized over sunscreen for primary prevention of skin cancer [30]. Observational studies have previously suggested that rates of sunburn are higher among those who report using sunscreen [31–33]. While this may be because individuals at greater risk for sunburn are more likely to use sunscreen, our data and others’ data suggest that people may also use sunscreen to prolong otherwise unprotected time in the sun. Shade is not waterproof and therefore cannot be worn during aquatic activities. We and others have reported that individuals are often near water or swimming at the time of sunburn [31, 34], suggesting the importance of having a device capable of monitoring sun exposure during aquatic activities. Finally, we did not observe differences in the use of the device or the effect of the intervention by age group, suggesting that the technology-based intervention was not more effective in younger participants. While there are gaps in the ownership of smart phones and use of other technology between young and older adults, these results are not surprising given one, the narrowing of this gap over the last decade [35] and two, the requirement of a smart phone for study entry.

The underlying idea of the intervention was that making people aware of their UVR exposure in real-time would motivate them to reduce that exposure. While unintentional UVR exposure more frequently leads to sunburn than intentional tanning, it is possible that promoting awareness is not sufficient to change action. Barriers to sun behavior changes may be higher in Northern climates where summers are short—for example, in Minnesota, where the study was conducted. Minnesota is second only to Utah in sunburn prevalence [36] and fourth highest for UVR-attributable incidence of melanoma [37]. These high rates can likely be traced to weather-related intermittent sun exposure and greater likelihood of sunburn, which increases melanoma risk, along with outdoor-focused summer and fitness lifestyles.

Finally, behavior change is difficult. Two other studies have aimed to improve sun protection behaviors (in addition to skin self-examination) among melanoma survivors, with varying levels of success. After an interactive online intervention, Manne et al. detected small improvements in sun-protective behaviors at 24 weeks [11]. Bowen et al. reported that following a web-based family communication intervention to increase self-skin examinations and sun-protective behaviors, some behaviors changed—wearing clothing and/or sunglasses, seeking shade, avoiding peak sun hours—but not sunscreen use [14]. Notably, this study reported low engagement in the intervention.

Strengths of the current study include a strong study design with rigorous implementation (blinded randomized clinical trial), successful participant recruitment despite study implementation during the COVID-19 pandemic, high participant retention, and collection of objective UVR data. Beyond the already described flaws of the device, including the need to back-transform data, limitations included the use of self-report outcome measures, which may be prone to social desirability bias in this study population. Further, with melanoma survivors as the participants, we acknowledge that their prior melanoma diagnosis and knowledge of the risk factors may affect their sun exposure behaviors and these results may have been different among participants without a history of skin cancer. While the study was conducted remotely, allowing it to proceed largely unimpeded through the COVID-19 pandemic, it is not clear how or to what extent people’s sun exposure may have been affected by the pandemic. We have no a priori reason to think that such effects would have been differential between the study arms, but this may limit generalizability of our findings in a post-pandemic era.

## Conclusion

We found no evidence that a UVR-exposure measurement intervention resulted in differences in sun protective or exposure behaviors, which may have been due, in part, to unexpected problems with the device. Based on our experience, and before further research is conducted with UVR-measuring devices similar to the one we used, the technology needs considerable improvement—for example, by integrating it into reliable devices people already use and by waterproofing the device. Additional changes to the mobile app messages are needed to promote more comprehensive sun protection behaviors, primarily sun avoidance and clothing [38], with sunscreen promotion only as a secondary measure to be used in conjunction with these more effective sun protection behaviors.

## Supporting information

S1 Checklist CONSORT 2010 checklist of information to include when reporting a randomised trial*. (DOC)

S1 Protocol (DOCX)
