# Supplementary material for: A UVR-sensor wearable device intervention to reduce sun exposure in melanoma survivors: Results from a randomized controlled trial
Source: PLoS One. 2023 Feb 10;18(2):e0281480. doi: 10.1371/journal.pone.0281480 (PMC9916644; doi:10.1371/journal.pone.0281480)
Supplement: S1 Protocol — (DOCX) [file pone.0281480.s002.docx]

| **Protocol Title** | Wearable device intervention to improve sun behaviors in melanoma survivors |
| --- | --- |
| **Principal Investigator/Faculty Advisor** | Name: Rachel I. Vogel |
|  | Department: Obstetrics, Gynecology, and Women’s Health |
|  | Telephone Number: 612-624-6928 |
|  | Email Address: isak0023@umn.edu |
| **Scientific Assessment** | Cancer Protocol Review Committee (CPRC) |
| **IND/IDE # (if applicable)** | N/A |
| **IND/IDE Holder** | N/A |
| **Investigational Drug Services # (if applicable)** | N/A |
| **Version Number/Date:** | Version 3.2 |

**PROTOCOL COVER PAGE**

**REVISION HISTORY**

| **Revision #** | **Version Date** | **Summary of Changes** | **Consent Change?** |
| --- | --- | --- | --- |
| 1 | 01/27/2020 | This revision incorporates a number of changes. We have added an Appendix at the end of the protocol (following the references; page 47) which summarize these changes and the rationale for each change. | Y |
| 2 | 03/05/2020 | We added the option to electronically sign the consent and HIPAA forms in REDCap given current COVID-19 health situation. Minor changes to exclusion criteria, recruitment, randomization stratification, and lottery procedures to accommodate requests from HealthPartners IRB. | N |
| 3 | 05/08/2020 | Clarifications of changes in study enrollment process due to COVID-19, all materials finalized. | N |
| 4 | 07/19/2021 | Increase to number of people to be contacted/invited to participate  Increase recruitment planned numbers to align with final recruitment numbers due to slightly higher than expected drop-out between verbal screening consent and written consent; and between written consent and baseline survey completion/randomization. | N |
|  |  |  |  |
|  |  |  |  |
|  |  |  |  |

Table of Contents

[1.0 Objectives 5](#_Toc528568482)

[2.0 Background 5](#_Toc528568483)

[3.0 Study Endpoints/Events/Outcomes 8](#_Toc528568484)

[4.0 Study Intervention 9](#_Toc528568485)

[5.0 Procedures Involved 13](#_Toc528568486)

[6.0 Data and Specimen Banking 15](#_Toc528568487)

[7.0 Sharing of Results with Participants 16](#_Toc528568488)

[8.0 Study Population 16](#_Toc528568489)

[9.0 Vulnerable Populations 17](#_Toc528568490)

[10.0 Local Number of Participants 18](#_Toc528568491)

[11.0 Local Recruitment Methods 18](#_Toc528568492)

[12.0 Withdrawal of Participants 19](#_Toc528568493)

[13.0 Risks to Participants 20](#_Toc528568494)

[14.0 Potential Benefits to Participants 20](#_Toc528568495)

[15.0 Statistical Considerations 21](#_Toc528568496)

[16.0 Confidentiality 23](#_Toc528568497)

[17.0 Provisions to Monitor the Data to Ensure the Safety of Participants 23](#_Toc528568498)

[18.0 Provisions to Protect the Privacy Interests of Participants 24](#_Toc528568499)

[19.0 Compensation for Research-Related Injury 24](#_Toc528568500)

[20.0 Consent Process 24](#_Toc528568501)

[21.0 Setting 24](#_Toc528568502)

[22.0 Multi-Site Research 25](#_Toc528568503)

[23.0 Resources Available 25](#_Toc528568504)

[24.0 References 40](#_Toc528568505)

**ABBREVIATIONS/DEFINITIONS**

- AUC Area under the curve
- CESR Center for Evaluation and Survey Research
- HIPAA Health Insurance Portability and Accountability Act
- ITT Intention-to-treat
- MI-MCMC Multiple imputation Markov Chain Monte Carlo
- QOL Quality of life
- UTAUT Unified Technology Acceptance and Use Theory
- UVR Ultraviolet radiation

# Objectives

- 1. Purpose:

Our purpose is to improve sun protection behaviors and reduce sun exposure among melanoma survivors by increasing their awareness of ultraviolet radiation (UVR) exposure by using a wearable UVR-sensor enabled technology device and corresponding mobile application designed to reduce sun exposure and promote healthy sun protection habits.

The Specific Aim of this study is to evaluate the effectiveness of a UVR-sensor wearable device intervention to improve sun protection behaviors and reduce UV exposure and sunburns in a randomized controlled trial in melanoma survivors.

# Background

- 1. Significance of Research Question/Purpose:

Melanoma, one of the most serious types of skin cancers, has been increasing in incidence over the past 30 years. With a 5-year survival rate of 93%, there are currently over one million melanoma survivors in the United States ^1^. Melanoma is considered a generally preventable cancer, with excessive UVR exposure being one of the strongest risk factors for the disease ^2,3^. Patients diagnosed with melanoma experience high rates of recurrence and second melanomas, with an approximately 9-fold increased risk of developing another melanoma ^4^. Patients who recur or have a second melanoma diagnosis have significantly worse prognosis ^5,6^. Importantly, UVR exposure following a melanoma diagnosis can be modified to reduce risk of a new melanoma diagnosis ^7^. Our recent study found that less than half of long-term melanoma survivors reported often or always staying in the shade or wearing a hat, and 20% reported a sunburn in the past year ^8^. Therefore, while some melanoma survivors are reporting healthy UVR exposure and protection behaviors, opportunities clearly remain to reduce future melanoma risk in the majority of melanoma survivors. Therefore, it is the purpose of this study to implement an intervention to promote healthy sun protection behaviors.

- 1. Preliminary Data:

Our preliminary data show the need for intervention in this population and highlight our extensive experience recruiting melanoma survivors, measuring sun exposure and protection behaviors in this population, and using technology devices to change health behavior.

In 2009, we completed the Skin Health Study (NIH/NCI R01), a population-based case-control study to assess use of indoor tanning, lifetime sun exposure, and use of sunscreen, statins and NSAIDs in relation to melanoma. The study included men and women, ages 25-59 at diagnosis or reference date (for controls). Cases were diagnosed with a first primary invasive melanoma between July 2004 and December 2007, identified by the Minnesota Cancer Surveillance System. Controls (individuals without melanoma) were randomly selected from the State’s driver license lists. A total of 1167 cases and 1101 controls completed a questionnaire about pre-diagnosis exposure (or reference date for controls) for lifetime exposure to solar and artificial UVR, skin, eye and hair color, freckles and moles, family history of skin cancer, lifetime UVR exposure, sunburns, and sunscreen use. The study resulted in seven co-authored papers ^9-15^.

We transitioned from the 2009 study to a series of studies to document the long-term effects of a melanoma diagnosis and treatment on survivors and compare their quality of life (QOL) and health behaviors with population controls. First, a series of focus groups were conducted to describe the breadth of experiences among melanoma survivors, focusing on the experience at diagnosis, ongoing physical, emotional and social concerns, and behavioral changes since diagnosis ^16^. The focus groups included 33 men and women diagnosed with Stage I-III cutaneous melanoma. Melanoma survivors participating in this study reported a diverse set of physical, emotional, and social concerns following diagnosis. They reported a high level of isolation and loneliness, underscoring the need to increase both awareness of melanoma and chances for melanoma survivors to interact and support each other. Almost 80% reported worrying about their melanoma reoccurring or a new cancer developing. Interestingly, while many reported increased sun protection behaviors, some reported no changes and others felt as though they did not know which changes to make.


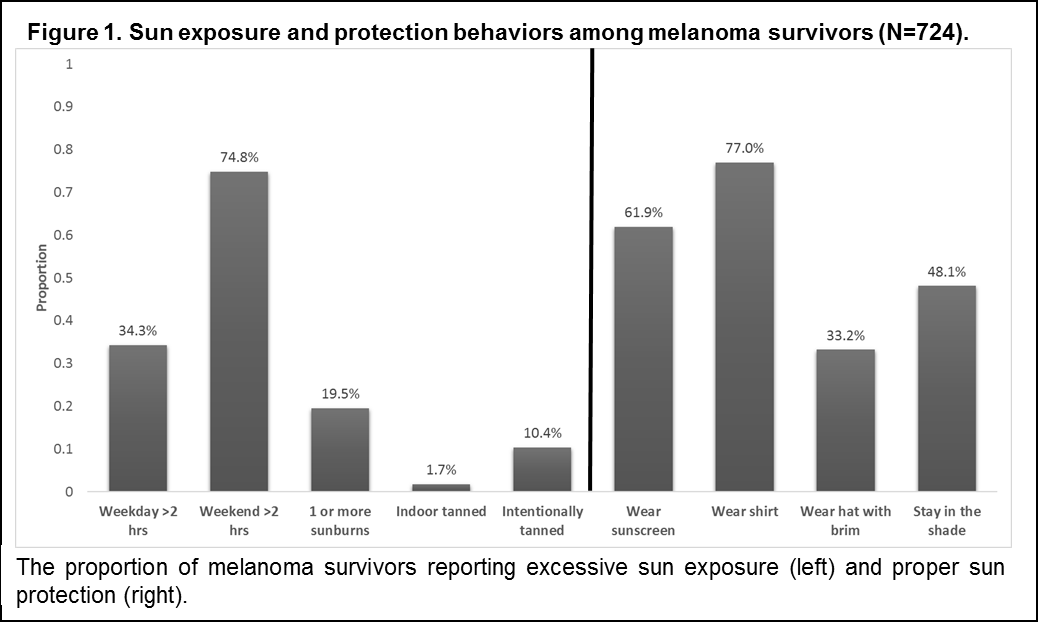
Based on these data, a comprehensive questionnaire to address QOL issues and health behaviors of melanoma survivors was designed and tested. Then a cross-sectional study of melanoma survivors and population controls was conducted. The goal was to collect data to evaluate the impact of melanoma on long-term survivorship focusing on quality of life ^17^ and sun exposure and protection behaviors ^18^. A total of 724 melanoma survivors and 660 controls from the parent study completed the survey. Melanoma survivors were diagnosed an average of 9.6 ± 1.0 years prior to completing the survey; the majority (85.6%) had stage I disease, 6.4% had stage II disease, 6.6% had stage III disease, and 1.4% had stage IV disease. The most common anatomic site of the tumor was the trunk (35.0%), followed by legs (26.9%), arms (26.4%), and head/neck (11.7%). Almost all (99.3%) had surgery and about one-third had lymph node dissection (34.8%). Melanoma survivors, compared to controls, reported statistically significant but only slightly higher physical functioning and bodily pain QOL subscale scores than controls and otherwise similar QOL as measured by the remaining six SF-36 subscale scores ^17^. Prevalence of anxiety and depression were similar between melanoma survivors and controls.

Our data regarding sun exposure and protection behaviors indicated that long-term survivors of melanoma reported greater use of sun protection behaviors than controls ^8^. Particularly important to this application, however, was the significant subgroup of melanoma survivors (20%) who experienced sunburn in the past year, reported intentional tanning (10%), did not wear a hat (67%) or stay in the shade (52%), and spent more than two hours in the sun on weekends (75%), putting them at elevated risk for future melanomas (Figure 1).

In addition to our expertise on melanoma survivors and sun protection, we also have conducted research in using technology to change behavior. In two large-scale federally funded research studies of physical activity (R01AG023410, R01-HL072947), and one large-scale federally funded study of weight-loss maintenance (R01-CA128211), collaborator B.C. Martinson employed electronic devices in the form of accelerometer-based actigraphy monitors for collection of objective measures of physical activity. He served as Co-Investigator on an industry-funded pilot project using Internet-enabled scales as weight monitoring and feedback devices to foster behavior change to support healthy weight loss (“Weigh By Day Pilot Study,” PI: Nico Pronk). The experience gained from these studies in overcoming challenges associated with the distribution to, and training of participants in the use of electronic monitoring devices, as well as data collected, data summarization and reporting will ensure the successful use of monitoring devices in the proposed study.

- 1. Existing Literature:

Previously, no interventions to promote healthy sun protection behaviors have been studied in melanoma survivors. Prior studies have typically focused on individuals at moderate to high risk of developing skin cancer but not melanoma survivors ^19-21^. One model for behavior change that has been effective in other areas of preventive health is the Fogg Behavior Model ^22^. According to this model, the drivers of behavior are: 1) motivation, 2) ability, and 3) triggers. Technology can be a useful tool to implement these drivers. New wearable technologies, which fall under the umbrella of ecological momentary interventions, incorporate UVR sensors to track exposure in addition to tracking other behaviors such as physical activity. This technology may be an important addition to melanoma prevention because prior exercise and activity interventions have not addressed sun exposure and protection and a recent analysis of national U.S. data suggest that higher physical activity is associated with sunburn ^23^.

# Study Endpoints/Events/Outcomes

- 1. Primary Outcome:

Sun protection habits index, measured using Glanz et al., 2008 questionnaire, will be scored by taking the average of 6 protective behaviors (wearing a shirt with sleeves, wearing sunglasses, staying in the shade, using sunscreen, limiting time in the sun, and wearing a hat) on a 4-point ordinal scale ranging from 1 = rarely or never to 4 = always. This has been used previously in similar intervention studies ^19^

- 1. Secondary Outcomes:
- Sunburn during the intervention period
- Each sun protection behavior separately
- Sun/UVR exposure (The device will provide objective measures of daily UVR exposure)
- Sun protection knowledge
- Unintended Consequences: we will measure physical activity, depression and anxiety to examine the potential unintended negative consequences of the intervention. Self-reported physical activity, along with other healthy behaviors such as fruit and vegetable consumption, will be ascertained using the American Health Association’s Life’s Simple 7 ^24^. Depression and anxiety will be measured using the Hospital Anxiety and Depression Scale (HADS) ^25^.

# Study Intervention

- 1. Conceptual Model and Description:

We are adopting the Fogg Behavioral Model ^26^ as our theoretical guide for this intervention, as depicted in the conceptual framework chart (Figure 2). Following the Fogg Behavioral Model, we will address barriers, examine motivators, and provide appropriate triggers to encourage optimal sun protection behaviors in melanoma survivors. The intervention will identify individual barriers that prevent melanoma survivors from optimal sun protection behaviors and then use messages (through website/brochure) to reduce perceived barriers and increase motivation to change. When people are highly motivated to change behavior and have the means to conduct the goal behavior, triggers are employed to prompt action; here, triggers would be messages regarding sun protection and exposure delivered via the wearable device as it detects UVR exposure.

**Figure 2. Conceptual Framework of the Study**


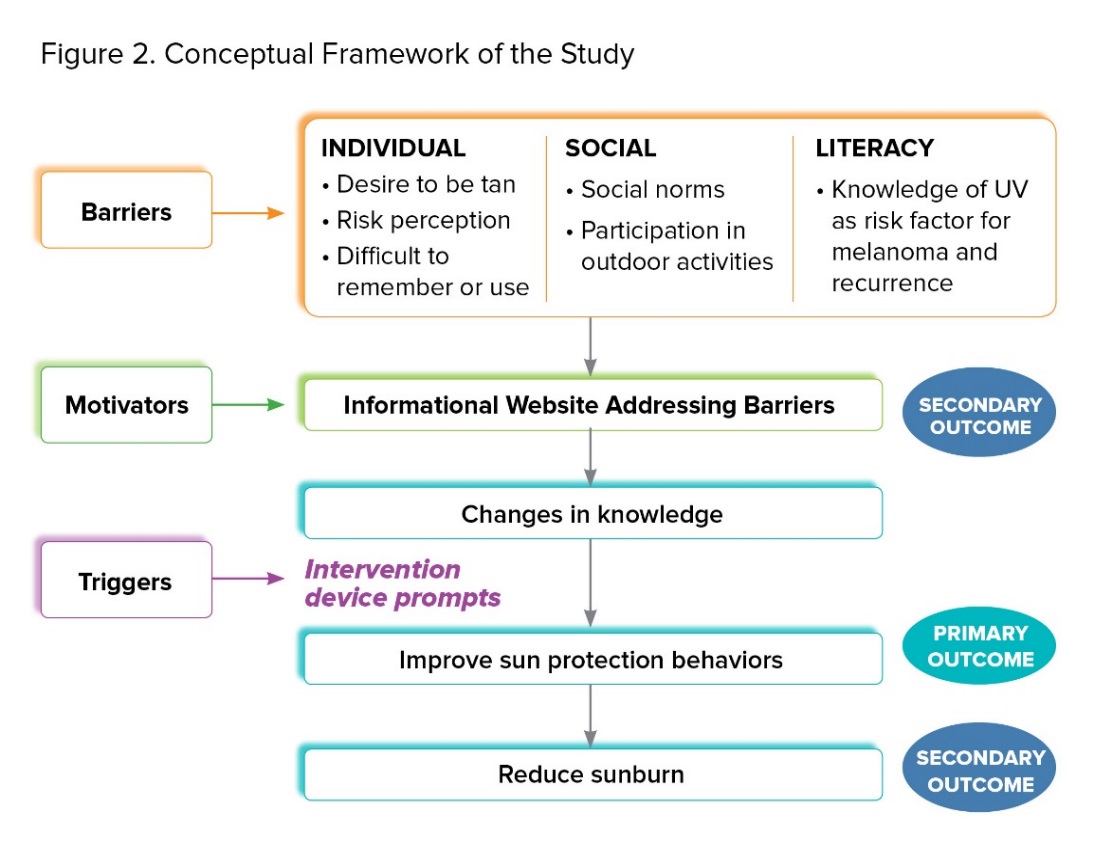

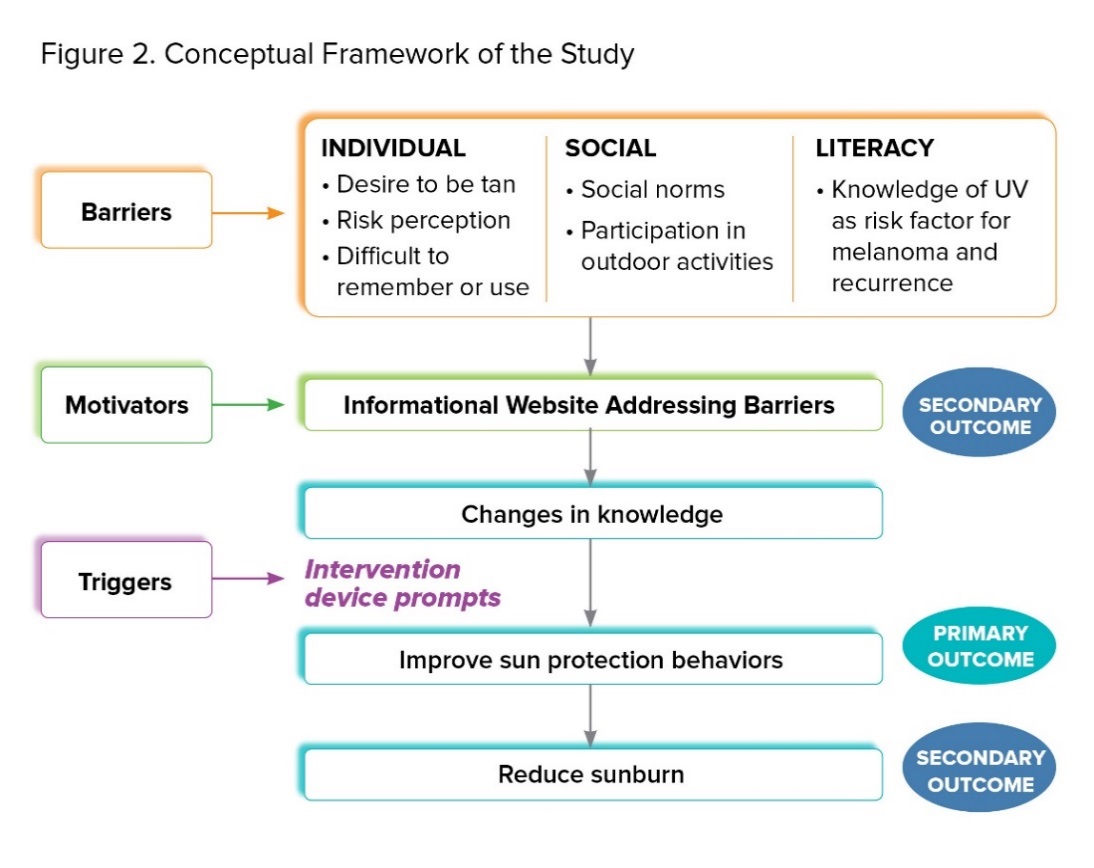

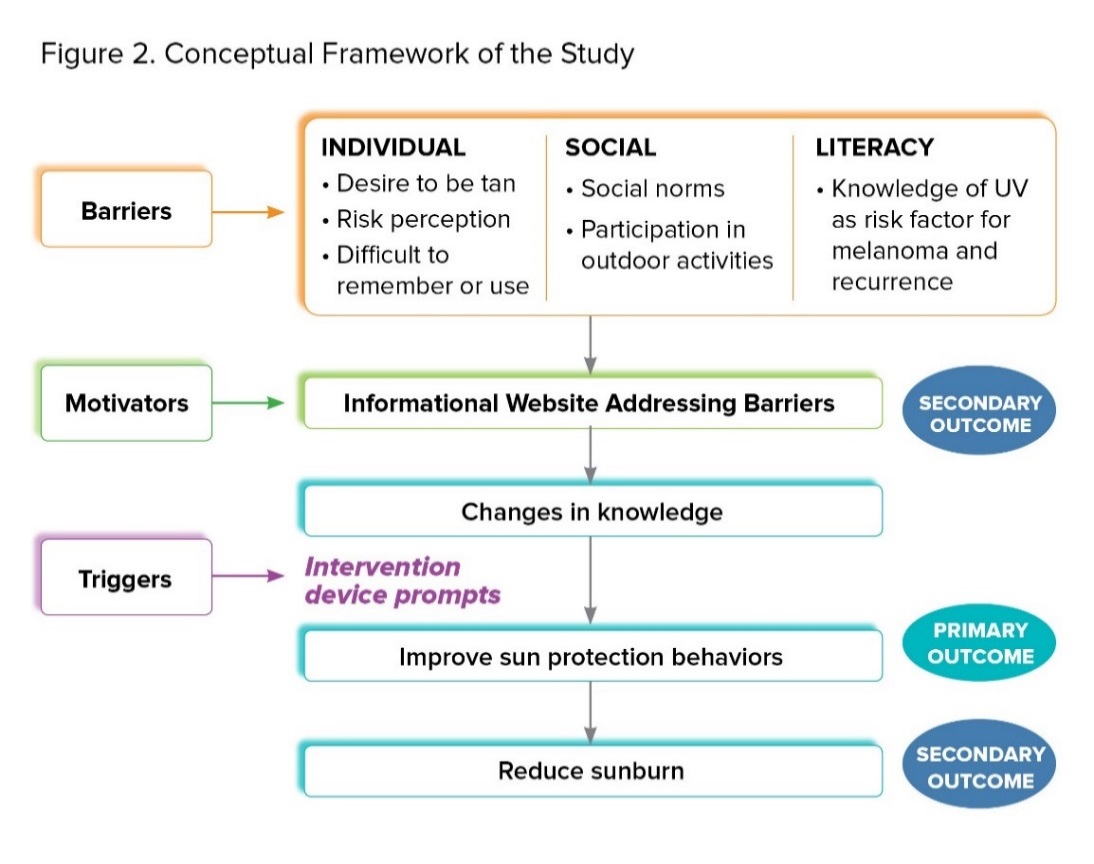


Figure 2 Conceptual Framework of the Study

Intervention Components:

We propose to use a wearable device that monitors UVR exposure to promote optimal sun protection behaviors. This intervention was chosen instead of an information-only website or mobile application because while knowledge is one component of behavior change, the intervention should address barriers, provide motivators and more importantly triggers to ultimately change behavior. In conjunction with the device and its associated mobile application, we will also develop a website that will provide information about the importance of UVR avoidance and protection in the population, in addition to supporting other health behaviors. Both intervention components will be provided to study participants randomized to the intervention group; the control group will received modified versions of each component.

(1). UVR-sensor enabled wearable band. We will provide a UVR-sensor enabled wrist wearable device to all participants. Shade, 2^nd^ generation, was developed in collaboration with the NIH National Cancer Institute. The researchers who developed this device have shown its UVR measurements to be significantly more accurate than other similar devices ^27^ and melanoma survivors reported it was easy to use.^28^ In addition to accurately measuring UVR exposure in a manner that does not rely on self-report and memory, Shade includes a mobile app that helps users set daily limits that take into account UVR intensity, time of exposure, and sunscreen application. Importantly, the app and device provide prompts when approaching the daily limit. The device will alert participants once they reach 20% of their daily limit (and every 20% after that) via a notification on the participant’s phone. It will also prompt them in the morning to put the device on and in the evening to place it on the charger.

(2). Informational Website. An organized library of information on melanoma and numerous health behaviors, including physical activity and sun exposure and protection, will be provided to participants in both groups. Participants will be sent a link to the website following completion of the online surveys during the intervention period. We will also include messaging based reported barriers to optimize sun protection (example messaging provided in Table 1) for those in the intervention group via the website and as a printed brochure which will be provided at baseline. The messaging will be pre-tested. While it has been widely reported that health education alone is insufficient to promote behavior change, a recent review of skin cancer knowledge and sun-related behaviors found that the majority of studies reported a positive association between sun protection behaviors and knowledge.^29^ Interestingly, however, the data did not support a relationship between knowledge and less tanning or sun exposure.

| **Table 1. Example messages to address perceived barriers to sun protection** | | |
| --- | --- | --- |
| **Perceived Barrier** | **Educational Message** | **Action Message** |
| “I want to be/look tan because it is more attractive.” | The sun causes more than 90 percent of the visible changes commonly attributed to skin aging ^30^. | Think about the feelings you associate with being tan? Are there other ways to generate those feelings without tanning? |
| “I feel left out if I’m not in the sun with my friends/family.” | People diagnosed with melanoma are at an approximately 9-fold increased risk of developing another melanoma ^4^. However, UVR exposure following a melanoma diagnosis can be modified to reduce risk of a new melanoma diagnosis ^7^. | Find an opportunity to educate your friends on melanoma and the importance of sun avoidance and protection for everyone. You may also consider a self-tanner or tanning lotion to make your skin look tanner. |
| “I worry about maintaining healthy levels of Vitamin D.” | The safest way to obtain vitamin D is through diet and supplements. | Check your Vitamin D levels on an annual basis and use supplements as directed by your provider. |
| “I don’t like the feel of sunscreen. And it is expensive anyway.” | The most effective sun protection methods are seeking shade and covering up. | Try a few alternative products to see if you can find one that has an acceptable texture and/or fragrance. You could also consider covering up with a hat or clothing instead. |
| “I don’t think the sun is what caused my melanoma.” | Approximately 86% of melanomas can be attributed to exposure to UVR from the sun ^31^. | Speak with your dermatologist about ways you can reduce your risk for recurrence. |
| “I’m too old for it to matter, I’m paying for things I did as a kid.” | Contrary to popular belief, only about 23 percent of lifetime exposure occurs by age 18 ^32^. | Set an example for younger people in your life by avoiding and protecting yourself from the sun. |
| “I forget about it as my diagnosis gets further in the past.” | The risks for a new melanoma after an initial diagnosis can continue for up to 20 years ^4^. | Make sun protection routine. For example, use a daily face or body lotion with SPF protection. |

Control Group. The control group will be offered both components (device and informational website), however, they will be provided a version of the mobile app that only collects the UVR exposure and does not provide notifications as they reach daily limits or summary information about exposure. The controls will also be provided basic information on sun exposure and protection recommendations as part of the website, however they will not receive the messaging regarding barriers. The attention-control group was chosen over no intervention for a few reasons; one, we can objectively measure UVR exposure in both groups; two, providing the device to the control group will help with recruitment, engagement and retention.

- 1. Drug/Device Handling:

The proposed research involves the use of a commercially-available environmental tracker for UV exposure. It is not a medical device.

- 1. Biosafety: N/A
  2. Stem Cells: N/A

# Procedures Involved

- 1. Study Design:

The RCT among 368 eligible melanoma survivors will be conducted in two Waves (two consecutive summers), with planned adjustments to improve recruitment, compliance and participant retention between study Waves, see Figure 3. Wave 1 may enroll up to 184 participants, though given logistical constraints due to working from home during the pandemic, fewer may be recruited, with the plan to obtain the full 368 by the end of Wave 2.


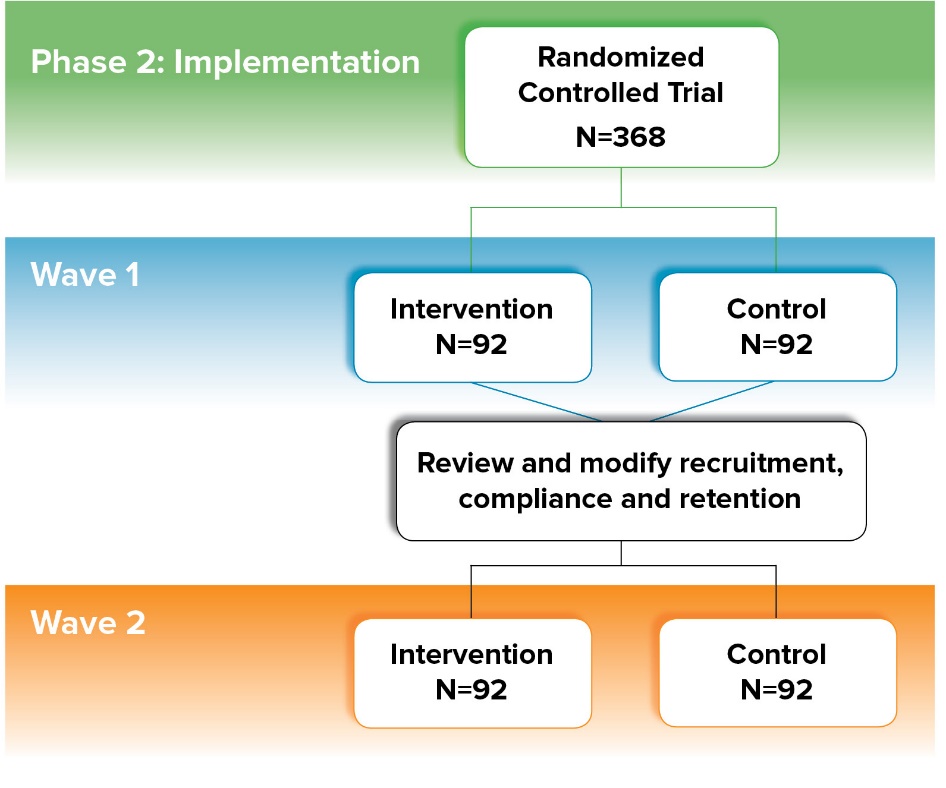


**Figure 3. Phase 2 Study Design.**

- 1. Study Procedures –Randomized Controlled Trial:

*Randomization.* Following written consent and completion of the baseline survey, participants will be randomized with a 1:1 ratio to the intervention or control arm. Randomization will be stratified by age (<50 years, 50+ years), gender, years since melanoma diagnosis (<2 years, 2-5 years, 6-9 years) and highest melanoma stage (I, II, III/IV). We will use block randomization with random blocks of size 2 and 4 to improve balance. The staff recruiting and enrolling participants will be blinded to their randomization. The study statistician will use R to generate random sequence numbers based on the stratified, block randomization scheme and upload the allocation list to REDCap. Following consent and screening, the study coordinator at the University of Minnesota will randomize participants.

*Study Initiation.* Following randomization, participants will then be mailed their device in early summer (with appropriate version of the mobile app based on randomization) and study assigned log-in information. This will include a study-created user name to be used with the device/mobile app to allow for direct retrieval of the device-collected data by the study coordinator. Participants will also be provided written training materials. Participants will be provided with a study phone number and email to contact the study coordinator with any remaining technical issues or questions.

*Study Procedures.* Users will be asked to wear the devices and sync it with the mobile app every day for 12 weeks, regardless of planned physical or outdoor activities. The study coordinator will view the data of all participants (both intervention and control) weekly to monitor device use. The study coordinator will contact participants who do not use their device more than 3 days in any given week to determine barriers and encourage use. Participants will be asked to complete periodic surveys and return the device via mail at the end of the intervention period.

*Data Collection.* Study measures will be collected via online surveys (in REDCap ^33^) and the Shade device. The survey will be accessible anywhere with an internet connection, see section 6.0. Self-administered questionnaire data will be collected at the start of the intervention (week 0), week 4, week 8 and week 12 (end of intervention), and week 64 (1 year after the end of the intervention; the end of the following summer). After completing the surveys, participants will be directed to the study website. Survey measures and their purpose are detailed in Table 2.

| **Table 2. Study Measures.** | | | | **Weeks** | | | | |
| --- | --- | --- | --- | --- | --- | --- | --- | --- |
| **Measure** | **Purpose** | **Description** | **# Items** | **0** | **4** | **8** | **12** | **64** |
| Health behaviors | Distraction | Measures of healthy behaviors, including alcohol, smoking, fruits, vegetables, vitamin D, height, weight | 10 | X | X | X | X | X |
| Physical activity | Secondary outcome  (unintended consequence) | Strenuous, moderate and mild activity times per week and amount of time per session | 6 | X | X | X | X | X |
| Sun Protection Habits | Primary outcome | Use of sunscreen, hat, shirt and shade while in the sun; Likert scale | 14 | X* | X* | X* | X* | X* |
| Sunburns | Secondary outcome (efficacy) | How many times did you have a red OR painful sunburn that lasted a day or more? | 1 | X** | X* | X* | X*,** | X** |
| Sun Exposure and other Protection measures | Validation/ Comparison with device data | Exposure during weekday and weekend days, sunscreen details, indoor tanning | 11 | X** | X* | X* | X* | X** |
| Beliefs, Intentions, Self-Efficacy regarding sun protection behaviors | Substudy (if intervention fails) | Reasoned Action Battery: Intention, Attitude, Behavioral beliefs, Normative pressure, Normative beliefs, Self-efficacy | 84 | X |  |  | X  (60) | X  (60) |
| Sun protection knowledge | Secondary outcome (efficacy) | Knowledge of sun protection (items covered in brochure) | 6 | X |  |  | X | X |
| Hospital Anxiety and Depression Scale | Secondary outcome  (unintended consequence) | Screener for depression and anxiety used frequently in cancer survivors; Likert scale | 14 | X |  |  | X | X |
| Comorbidities | Background |  | 14 | X |  |  | X | X |
| Diagnosis and treatment | Background/ Potential moderators | Year diagnosis, location, stage, treatments received, recurrence, metastasis | 18 | X |  |  |  |  |
| Fear of recurrence | Background/ Potential moderator | Cancer and health worry | 5 | X |  |  | X | X |
| Demographics | Background/ Potential moderators | Sex, age, race/ethnicity, education, income, marital status, parent status, family history of cancer, skin phenotype | 15 | X |  |  |  |  |
| Device use |  |  | 9 |  | X | X | X |  |
| Device satisfaction |  |  | 17 |  |  |  | X |  |
| Total number of items | | |  | **198** | **51** | **51** | **167** | **141** |

* Time frame: past 4 weeks ** Time frame: past summer

- 1. Study Duration:

While the entire study will take approximately 4 years in total, each participant will only be enrolled for a little longer than 1 year (12-week intervention implementation period, and follow up survey at 64-weeks).

- 1. Individually Identifiable Health Information:

HealthPartners will obtain patient name, address, email address, and telephone number based on melanoma diagnosis to contact potentially eligible participants. Among those who agree to participate in the study, we will obtain signed Health Insurance Portability and Accountability Act (HIPAA) authorization forms to seek data from HealthPartners and store information regarding their melanoma diagnosis, including date of diagnosis and disease stage. These data will supplement the self-reported data.

- 1. Use of radiation: N/A
  2. Use of Center for Magnetic Resonance Research: N/A

# Data and Specimen Banking

- 1. Storage and Access

All data (collected by the wearable device and survey data) will be stored in REDCap includes features to support HIPAA compliance with a full audit trail, user-based privileges, and integration with the institutional LDAP server. Only members of the research team will have access to the data.

Data collected by the wearable devices: The study coordinator will use the study user names to access the device use data via the Shade clinical trials dashboard. The data are maintained de-identified (study user ID only) indefinitely as part of the Shade dashboard and can be exported as a CSV or Excel file. The study coordinator will view the data of all participants (both intervention and control) weekly to monitor device use. Following the completion of the intervention period, the study coordinator will download the final device data which will be combined and merged with the REDCap survey data for statistical analysis.

*Survey data:* Survey data will be collected and stored in REDCap ^33^. The study survey will be accessible from any location with an internet connection. REDCap allows users to exit and return to the survey at any time without losing data. As the participant completes the questionnaire, the data are entered directly into the REDCap database, which uses a MySQL database via a secure web interface with data checks to ensure data quality.

- 1. Data:

# Self-administered questionnaire data will be collected at baseline, 4, 8, 12 and 64 weeks post-randomization (see Table 2 above).

# Demographics and Disease Information. Demographics will be ascertained at the baseline survey and will include age, race/ethnicity, income, education, employment status, relationship status, skin type and family history of cancer. Disease information will include disease stage, tumor location, treatments received and co-morbidities.

# Outcomes Measures. Outcomes will be measured via self-reported online surveys and the Shade device (see Section 3.0). Study outcomes include reliable and validated measures used previously by the study team and will be scored following standard procedures.

# Process Measures. We will focus on a number of metrics regarding study implementation, including recruitment, compliance/engagement, retention, and satisfaction/usability of the device. Recruitment metrics will include a proportion of eligible individuals who agree to participate and number of contacts required to recruit a participant. Measures of compliance and engagement will focus on device set-up and intervention usage, including time from recruitment to device use, average number of days the device is worn per week, number of times the study coordinator contacted to remind use, and self-reported engagement/response to the UVR notifications on the mobile app. Study retention will be measured as the proportion of participants who complete the baseline and each of the follow-up surveys. Finally, we will measure satisfaction with the device. The implementation metrics will be examined after Wave 1 is complete in order to identify areas of opportunity to improve recruitment, compliance/engagement and retention that can be implemented prior to starting Wave 2. While we will keep the intervention consistent across Waves, we will consider alternative methods of recruitment, incentives, reminders, and other actions to maximize engagement and retention.

- 1. Release/Sharing

Data collected in this study will not be released or shared to those outside of the research team.

# Sharing of Results with Participants

- 1. All participants (regardless of randomization group) will be provided a summary report of their personal sun exposure during the intervention period. Study results will be not be shared with the participants nor will study participation be shared with their providers. Participants will have access to their own data captured by the device via their mobile application.

# Study Population

- 1. Inclusion Criteria:
     - 18-75 years old
     - Diagnosed with cutaneous invasive melanoma within HealthPartners system since 2010
     - Able to read/write in English
     - Own a smartphone
     - Able to provide voluntary informed consent
  2. Exclusion Criteria:
     - Inability to provide informed consent
     - Pregnancy
     - Patients who have opted out of their records being used for research purposes
  3. Screening:

To identify eligible participants, HealthPartners medical claims data will be queried to identify members diagnosed with melanoma during the recruitment eligibility period (2010-forward). HealthPartners members who have a melanoma diagnosis (2 or more codes) between 2010 and the beginning of the study recruitment period will be queried from the VDW or ADW Administrative Claims database. If additional subjects need to be identified to meet recruitment goals, then HealthPartners or Park Nicollet patients who are not HealthPartners members will be queried from EMR data in the EPIC/Clarity database. ICD9 and ICD10 Codes of 172.xx and C43.xxx respectively will be used to identify eligible members or patients. Tumor registry data within the VDW may also be utilized to identify members or patients diagnosed with melanoma. Those identified as potentially eligible will be mailed an invitation letter from HealthPartners and contacted by phone to confirm eligibility.

# Vulnerable Populations

- 1. Vulnerable Populations:

| Population / Group | Identify whether any of the following populations will be targeted, included (not necessarily targeted) or excluded from participation in the study. |
| --- | --- |
| Children | Excluded from Participation |
| Pregnant women/fetuses/neonates | Excluded from Participation |
| Prisoners | Excluded from Participation |
| Adults lacking capacity to consent and/or adults with diminished capacity to consent, including, but not limited to, those with acute medical conditions, psychiatric disorders, neurologic disorders, developmental disorders, and behavioral disorders | Excluded from Participation |
| Non-English speakers | Excluded from Participation |
| Those unable to read (illiterate) | Excluded from Participation |
| Employees of the researcher | Excluded from Participation |
| Students of the researcher | Excluded from Participation |
| Undervalued or disenfranchised social group | Included/Allowed to Participate |
| Active members of the military (service members), DoD personnel (including civilian employees) | Included/Allowed to Participate |
| Individual or group that is approached for participation in research during a stressful situation such as emergency room setting, childbirth (labor), etc. | Excluded from Participation |
| Individual or group that is disadvantaged in the distribution of social goods and services such as income, housing, or healthcare. | Included/Allowed to Participate |
| Individual or group with a serious health condition for which there are no satisfactory standard treatments. | Included/Allowed to Participate |
| Individual or group with a fear of negative consequences for not participating in the research (e.g. institutionalization, deportation, disclosure of stigmatizing behavior). | Excluded from Participation |
| Any other circumstance/dynamic that could increase vulnerability to coercion or exploitation that might influence consent to research or decision to continue in research. | Excluded from Participation |

- 1. Additional Safeguards:

All participants will have had a melanoma diagnosis, which is a serious health condition. This study specifically aims to understand and intervene on sun risk behaviors among melanoma survivors. Therefore, melanoma survivors are the explicit target population of this study.

The proposed research will not specifically seek military members or DOD personnel, disadvantaged individuals or members of undervalued or disenfranchised social groups, however, if volunteers meets the inclusion criteria and also happen to be from one of the groups checked above, they will be eligible to participate in the study.

# Local Number of Participants

- 1. Local Number of Participants to be Consented as Eligible for Recruitment (Verbal): 460

Local Number of Participants to be Consented to Full Study (Written): 390

# Local Recruitment Methods

- 1. Recruitment Process:

Trained and experienced staff of HealthPartners Institute Center for Evaluation and Survey Research (CESR) will oversee the recruitment process. The CESR has been engaged in research both within the HealthPartners patient/member population and broader community for more than 15 years and is directed by a seasoned PhD survey methodologist and health services researcher, who oversees staff of bachelors and masters-level employees with experience and training in survey, interview, chart-audit, and electronic data capture for research purposes. The CESR employs REDCap (Research Electronic Data Capture) ^33^ as a primary data capture and management platform.

To recruit the required sample size and ensure meaningful measures of protection sun exposure, participants will be enrolled in two cohorts (Wave 1 and 2) over two consecutive summers. Recruitment will occur in the spring with the study intervention running over the summer both years to account for seasonality.

Potential participants identified by HealthPartners in the screening process will be randomly assigned to a mailing round (2 rounds of approximately n=500 each within each Wave). Each round will be mailed 2 weeks apart to ensure phone contact is proximal to the mail date. We will send potential subjects a letter on HealthPartners letterhead introducing the general nature of the study and the elements of informed consent, and notifying the participant that a study interviewer will soon call. Subjects will be provided a study phone-number, which they may contact if they would like more information about the study. The letter will state that the information obtained is confidential and that participation is voluntary. Next, within two weeks of sending each letter, an interviewer trained specifically on this study protocol will phone the subject to ask if he or she is willing to participate in the study if they have not opted out. The interviewer will obtain verbal consent to participate in the study and for their contact information (name, address, phone number) to be shared with members of the University of Minnesota research staff. If such consent is provided, it will be documented and their names and contact information will be so forwarded. All subsequent contact with these individuals in conjunction with this study will be conducted by study staff at the University of Minnesota

Once the goal of consenting participants has been reached for a given Wave, a wait-list will be created which will be used to replace individuals who do not complete the formal study consent process and baseline survey. Given our expectation of losing approximately 15% of individuals after this initial agreement, HealthPartners will provide the University of Minnesota with contact information for additional individuals.

The University of Minnesota staff will then contact individuals provided by HealthPartners by mail or email in spring to confirm their interest in participating in the study and obtain written or electronic informed consent and HIPAA forms along with their preferred form of contact for study communication.

- 1. Identification of Potential Participants:

To identify eligible participants, HealthPartners medical claims data will be queried to identify members diagnosed with melanoma during the recruitment eligibility period (2010-forward). HealthPartners members who have a melanoma diagnosis (2 or more codes) between 2010 and the beginning of the study recruitment period will be queried from the VDW or ADW Administrative Claims database. If additional subjects need to be identified to meet recruitment goals, then HealthPartners or Park Nicollet patients who are not HealthPartners members will be queried from EMR data in the EPIC/Clarity database. ICD9 and ICD10 Codes of 172.xx and C43.xxx respectively will be used to identify eligible members or patients. Tumor registry data within the VDW may also be utilized to identify members or patients diagnosed with melanoma. HealthPartners will be the only site of recruitment.

- 1. Recruitment Materials:

Participants will be mailed an introductory letter outlining the study purpose and providing an email address / phone number to contact the study coordinator if interested. HealthPartners will then follow-up by phone to obtain verbal consent to provide their contact information to the University of Minnesota. The University of Minnesota when then mail or email individuals an introductory letter and either paper consent and HIPAA forms (mail) or a link to access online consent and HIPAA forms. All recruitment materials are provided with the protocol.

- 1. Payment:

Participants will be provided gift cards by mail throughout the study period as they complete study procedures. They will receive $20 following completion of the baseline survey, $10 for the week 4 survey, $10 for the week 8 survey, $60 for returning the device at the end of the study period and completing the week 12 survey, and $20 for the week 64 survey (for a total of up to $120). In addition, a lottery-based incentive will be used to promote adherence. This incentive structure will indirectly support behavior change by incentivizing use of the devices rather than directly incentivizing the direct sun-exposure behavior. Participants will be entered into weekly lotteries for $100 gift cards for the duration of the intervention period (12 weeks; lotteries will be held up to 16 weeks given the rolling recruitment). The weekly winners will be randomly drawn from all participants. The randomly selected winners will only be eligible for the prize if they wore the device at least 5 days during that week, as evidenced by activity data. We will email the study group every week indicating that the lottery was drawn, a winner was chosen, and the number of draws required to obtain a participant who was eligible to win.

# Withdrawal of Participants

- 1. Withdrawal Circumstances:

No participant will be withdrawn from the study against their will. Participants will only be withdrawn from the study if they ask to withdraw or become too ill or die prior to completing the study. If participants become too ill, they will not be withdrawn from the study until they have given their voluntary consent for study withdrawal.

- 1. Withdrawal Procedures:

Participants that withdraw from the study for any reason will be noted in the REDCap study database and will no longer receive contact from study personnel. The data collected up until that point will be used in data analysis unless otherwise requested by the participant.

- 1. Termination Procedures:

Participants will not be terminated from this study without their consent as this is a randomized controlled trial and all participants are to be followed regardless of their device use.

# Risks to Participants

- 1. Foreseeable Risks:

This study carries minimal risk and will comply with the University of Minnesota IRB reporting requirements. We identified the following 2 possible risks to subjects:

Risk to confidentiality: Participants will be asked to keep their mobile phone password protected to guard their confidentiality. Inadvertent breaches of confidentiality by investigators or their staff are unlikely but may occur.

Identifying information will be kept private and all identifiers will be removed prior to any data being given to researchers. The records will be identified only with a unique ID number on an encrypted database. Data transfer will only occur with de-identified data with encrypted transfer of all information containing protected health information between participants and study databases.

Discomfort with survey questions: There is a possibility that some participants may feel uncomfortable being asked questions about their melanoma diagnosis and health behaviors.

- 1. Reproduction Risks: N/A
  2. Risks to Others: N/A

# Potential Benefits to Participants

- 1. Potential Benefits:

There are likely no direct benefits to individual participants. Individuals in both groups may increase their activity level during the study period due to prompting from the device. Should the intervention be effective, participants may increase their awareness about melanoma and risk factors for a second melanoma and may decrease their own risk through improved UVR exposure protection habits.

# Statistical Considerations

- 1. Data Analysis Plan:

# All questionnaires have been previously developed and implemented and will be scored using standard methods.

- 1. Power Analysis:

Anticipating 85% of those recruited to the study complete the 12 week follow-up survey, a sample size of N=314 participants with outcome data is expected. A sample size of 314 will achieve 95% power to detect an effect size of 0.40 between intervention and control groups using a two-sided two-sample t-test assuming a significance level of 0.05. The effect size is similar to that found in an intervention to promote sun protection behaviors in high-risk individuals with the same primary outcome ^19^. Importantly, it will also achieve 70% power to compare report of sunburn during the intervention period between the two groups (detect 20% of the control participants reporting a sunburn – as seen in our preliminary data - and 10% of the intervention participants reporting a sunburn) as statistically significant at 0.05 significance level.

- 1. Statistical Analysis:

# Statistical analysis will focus on evaluating the efficacy, unintended consequences and implementation of the intervention in this RCT. The main analysis of the primary outcome (self-reported overall sun protection score) will be a two-sample two-sided two-sample t-test comparing scores at the end of the intervention period (12 weeks) between intervention and control groups. Randomization should result in two groups that are similar for patient characteristics and other extraneous factors that may influence sun exposure and protection behaviors, however, we will compare the randomized groups by Wave, gender, age, stage of disease, device use (low/high), and baseline measures of sun protection and exposure behaviors using two-sample t-tests and Chi-squared tests to identify potential confounding factors which may not be balanced between the two groups. We will supplement the primary analysis with a multivariate linear regression model to account for any identified differences at baseline. A secondary analysis will explore changes in the sun protection score over the intervention period (baseline, weeks 4, 8 and 12) using a mixed effects regression model. This analysis will not be completed until both Waves have been conducted.

# Secondary analyses will focus on each self-reported sun protection behavior separately, sunburns, intentional tanning, skin cancer knowledge, and physical activity and health behaviors by comparing the intervention and control participants over the study intervention period and 1 year post-completion. Analyses will be conducted using Chi-squared and t-tests as appropriate for univariate analyses at each time point and logistic and linear regression models for multivariate analyses. Daily UVR data (minutes of UVR exposure per day) will be summarized using the area under the curve (AUC) over the 12 week intervention period. Proper transformation (e.g. log-transformation) will be applied if the AUC data are skewed. The two groups’ AUC will be compared using similar methods as proposed above. The association between sun exposure and its potential moderating effect on sun protection behaviors during the intervention will be investigated using linear mixed effects models including main and interaction effects, adjusting for potential confounders. To address any potential intervention effects in the control group, we will compare the baseline and post-intervention sun protection behaviors scores using paired t-tests, stratifying by randomization group. The proportions of survivors in the intervention and control groups with potentially clinically significant depression and anxiety will be compared using Chi-squared tests and supplemented with multivariate logistic regression models. Finally, descriptive statistics will be used to summarize the implementation metrics both across and within randomization group both between Waves 1 and 2 and after completion of the study.

# We plan to perform all primary statistical analyses following intention-to-treat (ITT) procedures. That is, all subjects allocated to a treatment or intervention will be followed up, evaluated, and analyzed as members of that group or treatment arm regardless of their compliance with the assigned treatment. While it is not expected that participants will be incorrectly assigned, some may not use the device and certain features. For that reason, we will also summarize device use and consider sensitivity analyses exploring the intervention effect using per-protocol methods (incorporating device use) as opposed to ITT. A priori, we anticipate that the amount of use of the intervention will predict sun protection and exposure behaviors, though we will likely be underpowered for this analysis.

# Every effort will be made to encourage participants’ compliance and data completeness. Nevertheless, we expect some amount of missing data. We plan to perform a comprehensive missing data analysis employing statistical methods that are valid under different missingness assumptions to determine if conclusions are sensitive to missing data. The primary missing data method will be multiple imputation using the Markov Chain Monte Carlo (MI-MCMC) method ^36,37^ assuming that data are missing at random—that is, the probability that an observation is missing can depend on the observed data but not on the missing data. We will include appropriate baseline variables into the MCMC procedure to make the conditional independent missing assumption less stringent. If the treatment group is associated with missingness, we will conduct multiple imputation for each treatment group separately ^38^. The MI-MCMC method allows arbitrary missing patterns including intermittent missing and dropout. We will also perform a simple imputation analysis as a sensitivity analysis; specifically, the linear interpolation method will be used for intermittent missing (e.g., when people forget to charge or wear the device), while the last week average value carried forward method will be used for dropouts, assuming that the average of the last 7 days’ data will be a good estimate of the missing data.

- 1. Data Integrity:

Data will be entered and stored in REDCap. Data will be reviewed for completeness and cleanliness on an ongoing basis by the study coordinator and statistician.

# Confidentiality

- 1. Data Security:

All study investigators and staff will be fully trained on data safety and participant confidentiality using appropriate HealthPartners, CITI and University of Minnesota courses. An electronic copy of the signed consent and HIPAA forms will be stored on an AHC-IS supported server and the paper copy (if collected) will be stored in a locked file cabinet in a locked office. The consent form and other research information will NOT be placed in the participants’ medical record.

Electronic data will be entered by the study coordinator and will be stored on the secure REDCap database. Study data will be de-identified before data analysis. Only the researchers directly involved with the study will have access to the data. Identifying data will be stored until completion of the study and manuscript submission.

# Provisions to Monitor the Data to Ensure the Safety of Participants

- 1. Data Integrity Monitoring:

The PI will review all signed consent and HIPAA authorization forms for completeness at the time of participant entry into the study.

Apart from the UVR and physical activity data that are automatically collected by the wearable devices, data collection will occur primarily using REDCap, which will be set up to ensure data are clean and ready for analysis. Reports within REDCap will be used to identify missing data.

- 1. Data Safety Monitoring:

This study carries minimal risk and therefore the PI will assume responsibility for monitoring and reporting safety concerns/events to the University of Minnesota IRB. Events requiring prompt reporting include any adverse event that requires a change to the protocol or consent form, any unauthorized disclosure of confidential information, any unresolved subject complaint or any protocol deviation that resulting in harm or the unanticipated death of an enrolled subject.

Any event requiring prompt reporting to the IRB will also be reported to the Masonic Cancer Center’s SAE Coordinator (email - [mcc-saes@umn.edu](mailto:mcc-saes@umn.edu)).

# Provisions to Protect the Privacy Interests of Participants

- 1. Protecting Privacy:

Identifying information will be kept private and secure as described above. Identifiers will be removed prior to data analysis. The records will be identified only with a unique ID number on an encrypted database. Data transfer will only occur with de-identified data with encrypted transfer of all information containing protected health information between participants and study databases.

Participants will be reminded they do not need to answer any survey questions they do not want to and that they can withdraw from the study at any point.

- 1. Access to Participants*:*

All participants will be asked to provide a signed HIPAA authorization form indicating their agreement for the research study team to access their medical records pertaining to their melanoma diagnosis and treatment.

# Compensation for Research-Related Injury

- 1. Compensation for Research-Related Injury: N/A
  2. Contract Language: N/A

# Consent Process

- 1. Consent Process:

Consent and HIPAA forms to participate in the RCT will be part of the initial recruitment mailing package from the University of Minnesota. The current plan is to include a self-addressed envelope for participants to return signed consent forms back to the study team, however, we have also planned to convert to email invitation and electronic consent given the COVID-19 health crisis and the potential for needing to coordinate the study entirely online. If a participants requires more information, they can state that before deciding whether or not they would like to participate in the study, and the study will be explained in more detail.

Consent and HIPAA forms will be stored in a secure file cabinet in a locked office in addition to being scanned and stored on password-protected secure servers. Online consents and HIPAA forms will obtain signatures captured electronically via the e-consent functionality in REDCap. At the bottom of the e-consent participants are asked whether they wish to receive signed copies or opt out of receiving them. Those wishing to receive them will provide their mailing address to receive paper copies through the USPS mail. Additionally, participants will also have the option to download signed copies of these documents to their own computer or electronic device directly from REDCap.

Electronic signatures will be stored within REDCap as well as a copy on the Academic Health Center (AHC) secure servers. Access to these records will be limited to the study team as needed or required.

- 1. Waiver or Alteration of Consent Process (when consent will not be obtained): We will request a waiver of consent through HealthPartners to access medical records to identify members (and patients if needed) eligible for the study who will be contacted via mail and phone by HealthPartners.
  2. Waiver of Written/Signed Documentation of Consent (when written/signed consent will not be obtained): HealthPartners will be requesting a waiver of documentation of consent for written consent for permission to provide contact information to the University of Minnesota. Recruited individuals will provide verbal consent via their recruitment phone call to proceed to study enrollment (written consent through University of Minnesota).
  3. Non-English Speaking Participants: N/A
  4. Participants Who Are Not Yet Adults (infants, children, teenagers under 18 years of age): N/A
  5. Cognitively Impaired Adults, or adults with fluctuating or diminished capacity to consent: N/A
  6. Adults Unable to Consent: N/A

# Setting

- 1. Research Sites:

The research will be conducted by the University of Minnesota and HealthPartners. This study will have a single recruitment site as we will only recruit individuals through HealthPartners members (and potentially patients). Participants can fill out the study survey from any location with online access. Both sites will conduct their own scientific and ethics review of the protocol and provide documentation of approval.

- 1. International Research: N/A

# Multi-Site Research

# N/A

# Resources Available

- 1. Resources Available:

Funding: This study will be conducted with grants to R.I. Vogel from the American Cancer Society and Melanoma Research Alliance.

Research team: All investigators are committed to this project and will provide appropriate effort as needed. Dr. Vogel will have a study coordinator who will be supported 50% time to conduct the study.

Participant Availability: HealthPartners is the largest consumer-governed nonprofit health care organization in the country, providing care, coverage, research, and education to improve health and well-being in partnership with its members, patients and community. Founded in 1957, the HealthPartners family of care serves more than 1.5 million medical and dental health plan members and more than 1 million patients. The organization includes a multispecialty group practice of more than 1,700 physicians; 7 hospitals; 47 primary care clinics; 22 urgent care locations; 22 dental clinics; and numerous specialty practices in Minnesota and western Wisconsin. Given very low levels of uninsured in Minnesota (single-digit percentages over the past decade), high population penetration of managed care in the state, and a high health insurance market-share, the HealthPartners member and patient populations are generally representative of the larger population.

Using data from HealthPartners 2010-2016, we expect over 2000 members will meet our eligibility criteria. We have previously found melanoma survivors to be highly motivated to participate in research, with participation rates of 70-85% for interviews and focus groups. While this is a more intensive study, we anticipate no issues recruiting 248 individuals.

The research outlined in this protocol was presented to the Cutaneous Oncology Translational Working Group in December 2017 and was met with enthusiasm from the dermatologists, surgeons, and oncologists treating patients with melanoma at the University of Minnesota. They verbally agreed to support this study and should recruitment by HealthPartners become a concern, University of Minnesota/Fairview patients may be considered.

**UNIVERSITY OF MINNESOTA**

The University of Minnesota is one of the state’s greatest assets. It is one of the most comprehensive universities in the United States and ranks among the most prestigious. The University, with more than 370 fields of study, offers more choices and unique opportunities for its 60,000 students. It is both the state land grant university and Minnesota’s only research university, where new knowledge, new products, and new services improve the quality of life for all Minnesotans. Another major resource is the University of Minnesota’s research activities. The University is one of the leading recipients of federal research awards. The University received more than $520 million in grant and contract awards from federal, state, and private sources in fiscal year 2010 and ranks in the top 10 of all public and private research universities. The University conducts 98 percent of all sponsored academic research in Minnesota.

Some of the many major resources at the University of Minnesota include an extensive library system. The University of Minnesota Libraries is one of the University's and the state's greatest intellectual and capital assets. Housed in 14 facilities on the three Twin Cities campuses, the University Libraries' collections contain 6.2 million print volumes, nearly 37,000 serial subscriptions, 6.3 million microforms, 2.65 million government documents, and 423,000 maps, making it the 16th largest research library in North America. Finally, the University of Minnesota has taken an aggressive, proactive stance in creating an atmosphere that is compliant with the Health Insurance Portability and Accountability Act (HIPAA). All personnel with any access whatsoever to human subject data, including custodians, are required to take a 3-part HIPAA training session. In addition, all individuals who work directly with human subjects data are required to take a course in protection of human subjects and all key personnel are required to take a 2-part course on the responsible conduct of research.

For this proposal, two research team members have academic homes in the Medical School, specifically, the Division of Gynecologic Oncology in the Department of Obstetrics, Gynecology and Women’s Health (Vogel) and Department of Dermatology (Ahmed-Saucedo), two members are located in the School of Public Health, including the Division of Epidemiology and Community Health (Lazovich) and the Division of Biostatistics (Luo), and one member is located in the School of Journalism and Mass Communication (Nagler). The resources and environment for each School are described below. All University of Minnesota research team members are also members of the Masonic Cancer Center.

**MEDICAL SCHOOL**

**Department of Obstetrics, Gynecology and Women’s Health**

The department is a nationally recognized entity that maintains several highly productive established programs while regularly adding new initiatives. Today the department hosts three very active academic divisions: Gynecology Oncology, Maternal Fetal Medicine and General Gynecology. On an annual basis, The Department of Obstetrics and Gynecology successfully compete for significant Federal and private funding. These endeavors regularly involve partnerships with other units within the Academic Health Center such as the National Center for Excellence in Women’s Health.

The Division of Gynecologic Oncology is heavily engaged in funded laboratory and clinical research. All faculty members are members of the Comprehensive Cancer Center and receive additional support from an NCI Cancer Center Support Grant. Support for the Division’s extensive research activities is also provided by peer-reviewed national non-profit organizations such as the Minnesota Medical Foundation, the Gynecologic Cancer Foundation (GCF), and a variety of other philanthropies and individual donations. Currently, the faculty has been awarded grants with total annual directed funds of more than $1,200,000 and 6 industry-sponsored studies exceeding $1,500,000.

**Office:** Dr. Vogel has a private office in the Division of Gynecologic Oncology on the 12th floor of Moos Tower (Room 12-262) at the University of Minnesota. The 12th floor also provides space for research staff to work. Each desk includes a desktop computer and/or a network connection for a laptop. Two conference rooms are available on the same floor for group meetings.

**Computer Services:**Dr. Vogel has a personal computer linked via Ethernet to a secure University network hosted by the Academic Health Center Information Services (AHS IS). The group supports databases, applications, web sites, file storage, and development of new tools.

**Data Management and Analysis Software**: Dr. Vogel has access to and regularly uses the Microsoft suite (Word, Excel, PowerPoint, and Access), SAS, PASS 14, R, and Prism. The University of Minnesota supports the use of REDCap, a web-based data collection system, for its researchers through the Clinical Translational Research Institute (http://www.ctsi.umn.edu/research/tools-software/REDCap/).

**Support Services**: The Department of OB/GYN works with the Department of Pediatrics to employ full-time staff of professional administrators to ensure that sponsored research projects, including research budgets, are appropriately managed and receive oversight to ensure responsible use of funds from sponsored research. These staff include an executive administrator, an office manager, a team of accountants, pre- and post-award grants managers and administrative assistants. These local services are supported by centralized university services including the Sponsored Projects Administration (<http://www.ospa.umn.edu/>) and the Institutional Review Board (http://www.research.umn.edu/irb), which are housed within the Office of the Vice-President for Research (OVPR) (<http://www.research.umn.edu/>). The University has numerous policies established for the protection of research subjects and the responsible management and oversight of the research conducted by University employees. These policies are available through the OVPR website.

**DEPARTMENT OF DERMATOLOGY**

On February 7, 1913, the Division of Dermatology and Genito-Urinary Diseases was created in the Department of Surgery at the University of Minnesota, and in 1914, Dermatology became a division in the Department of Medicine. In 1971, with the return of Dr. Robert W. Goltz from the University of Colorado to the University of Minnesota, Dermatology became a Department. Over 30 active faculty provide care and research in five divisions at the University site. These divisions include General Dermatology, Dermatologic Surgery, Pediatric Dermatology, Clinical Research and Dermatopathology. The teaching program is based at four teaching sites – University of Minnesota Medical Center, Fairview, Veterans Affairs Medical Center (VA), Hennepin County Medical Center, Park-Nicollet/Health Partners and the Children’s Hospital. Twenty-three residents rotate through these clinical sites in either categorical, internal medicine/dermatology or 2+2 residency programs. In addition to the dermatology residency programs, two fellowships are provided - in Pediatric Dermatology and Procedural Dermatology. Research is conducted primarily at the University and the VA sites and is collaborative and inter-disciplinary with faculty also associated with the Center for Immunology, Department of Pediatrics, Melanoma and Skin Cancer Program and the Clinical and Translational Scientific Institute (CTSI). Faculty are active in both local and national organizations including the Association of Professors of Dermatology, the Society for Investigative Dermatology and the American Academy of Dermatology. Faculty are also active members of national committees or boards including the NIH Arthritis, Connective Tissue and Skin Study Section and the Board of Directors of the National Alopecia Areata Foundation and Cicatricial Alopecia Research Foundation.

Dr. Ahmed has office space in the Department of Dermatology and participates regularly in the Department’s Clinical Research Division’s grant related activities. The Dermatology Clinic is located in the Clinics and Surgery Center (CSC). The University of Minnesota Clinics and Surgery Center is a new, 342,000-square-foot care facility that opened in February 2016 on the East Bank of the University of Minnesota Medical Center campus.

**SCHOOL OF PUBLIC HEALTH (SPH)**

The University of Minnesota School of Public Health (SPH) was founded in 1944 and provides graduate-level training in Public Health for scholars and health professionals from around the world. SPH is consistently recognized as one of the top public health programs in the country. SPH has 135 core faculty members across four academic divisions (Epidemiology and Community Health, Biostatistics, Health Policy and Management, and Environmental Health) who teach and conduct research in populations on disease prevention and health promotion.

**SPH - Division of Epidemiology and Community Health**

The Division of Epidemiology and Community Health (EpiCH) is the largest of four divisions in the School of Public Health. For over 70 years, EpiCH has led regional, national, and international studies of the distribution, causes, and prevention of major diseases. The signature EpiCH project is the ambitious and groundbreaking Seven Countries study (<http://sph.umn.edu/about/history/halfcentury>) led by public health pioneer Ancel Keys, which accelerated the prevention and treatment of cardiovascular disease and popularized the Mediterranean Diet.

EpiCH has 43 primary faculty members with diverse backgrounds and program emphasis in Clinical/Biological Sciences and Social/Behavioral Sciences. Current EpiCH faculty members are among the leading researchers in the world. In addition to all of the substantive research areas, EpiCH has a core set of faculty who work extensively in epidemiologic methods (<http://www.sphresearch.umn.edu/epi/epidemiologic-methods>) with specific expertise in mixed model regression, longitudinal data analysis, and latent variable modeling.

EpiCH faculty collectively have been awarded between $30 and 50 million annually in sponsored funds over the past decade to support ongoing research, the majority of which are competitive grant awards from NIH. As a function of the Division’s focus on extramural sponsored research, EpiCH Division Head Dr. Dianne Neumark-Sztainer has created a formal mentoring program to foster relationships for the express purpose of promoting grant writing and research implementation skills among junior faculty members by drawing upon the considerable expertise and success of experienced senior EpiCH faculty. The Division fosters regular communication among faculty members to provide peer support for all phases of the research process.

Dr. Lazovich has dedicated office space and support from Division administrative staff.

**SPH - Division of Biostatistics**

The Division of the Biostatistics at the University of Minnesota ranks consistently in the top eight of biostatistics programs in the United States. They have played a leadership role in many national and international clinical trials, including the first vaccine trial for Ebola and the largest HIV/AIDS treatment trial in history.

The Division of Biostatistics focuses on the development of statistical methods for biomedical research. They have faculty who specialize in numerous areas and collaborate with University research partners on projects involving HIV/AIDS, heart and lung disease, cancer, neuroimaging, and many other clinical disciplines. Performing methodological research in areas including Bayesian analysis, spatial statistics, statistical genetics, and causal inference, division faculty, students, and staff are involved in more than 100 research grants and contracts totaling more than $150 million.

Dr. Luo has dedicated office space and support from Division administrative staff.

**SCHOOL OF JOURNALISM AND MASS COMMUNICATION**

First established as an academic department in 1922 and housed in Murphy Hall since 1939, the University of Minnesota’s School of Journalism and Mass Communication is one of the oldest programs in the United States. Murphy Hall underwent a $10.5 million renovation in 2000, thus creating a state-of-the art facility for research and teaching in the areas of journalism, mass communication, and new media studies. Roughly 1,000 undergraduates pursue a major or minor in the program every year, and approximately 100 graduate students pursue MA and PhD degrees in Strategic Communication or Mass Communication.

The School of Journalism and Mass Communication benefits from state-of-the-art research, media production, and classroom facilities. Research facilities include the Communication Research Division Laboratory, which provides dedicated space for focus group and message testing research. Occupying approximately 400 square feet on the third floor of Murphy Hall, the space includes a reception area, focus group suite (including an observation control room), three participant testing rooms, and kitchen facilities. Additional features of the lab include an eye-tracking machine for use in message testing research, as well as a mobile game cart with game-playing devices for use in game simulation research. As a member of the Communication Research Division, Dr. Nagler has full access to the lab and its resources.

The School of Journalism and Mass Communication is also a stakeholder in the University of Minnesota’s Usability Services Lab, housed in the Walter Library Digital Technology Center. This lab provides technology and space for researchers to conduct usability testing (for example, eye-tracking studies of website navigation) and media effects research. In addition, as a faculty member in the College of Liberal Arts, Dr. Nagler has access to the University of Minnesota’s Social and Behavioral Sciences Laboratory (SBSL). The SBSL allows researchers to run multiple research subjects simultaneously, each at their own computer. These facilities are supported by a lab manager, lab technician, and research-computing consultants from the College of Liberal Arts’ Office of Information Technology. Academic oversight is provided by College of Liberal Arts Associate Dean Dr. Alexander Rothman. The SBSL offers the following resources: 44 networked workstations with Windows XP, 19-inch LCD screens, high-quality headphones, 22 serial response boxes, 22 microphones, and custom-configurable privacy carrels for all workstations. Data security in the SBSL is provided by a custom firewall for the lab network and optional encryption of internal network traffic. Pre-installed experiment software packages E-prime and Presentation are available to all researchers; custom configurations can be arranged.

Beyond its research facilities, Murphy Hall has myriad media production resources. Located on the ground floor of Murphy Hall, the Digital Media Studios is a newsroom-like media laboratory that has capability for video editing; digital and analog audio; and imaging, graphics, and communication design. It also includes a television studio. The Digital Information Resource Center and Sevareid Library, located next to the Digital Media Studios, is equipped with traditional and digital information resources for use in research and teaching, and includes eight networked workstations and two teaming rooms with digital editing capability.

Dr. Nagler has a permanent faculty office in Murphy Hall. Support staff include two full-time computer system specialists, who provide network and programming support to faculty.

**MASONIC CANCER CENTER**

The Masonic Cancer Center serves to advance knowledge by creating a collaborative research environment focused on the causes, prevention, detection, and treatment of cancer; applying that knowledge to improve quality of life for patients and survivors; and sharing its discoveries with other scientists, students, professionals, and the community. It was founded in 1991, and is part of the University's Academic Health Center, which also includes the Medical School, Dental School, College of Pharmacy, and Schools of Public Health and Veterinary Medicine. The Masonic Cancer Center includes more than 500 faculty and staff members. It is home to some of the world's top cancer researchers in bone marrow transplantation, breast cancer, bone cancer, cancer genetics, tobacco research, immunology, new therapies development, pediatric oncology, chemoprevention, and epidemiology. The National Cancer Institute (NCI) designated the Masonic Cancer Center, University of Minnesota a comprehensive cancer center in 1998, and in 2003, 2009, and 2013, NCI renewed this designation. The Masonic Cancer Center is one of only 41 institutions in the United States to hold this designation. It is awarded only to institutions that make ongoing, significant advances in cancer research, treatment, and education. The Masonic Cancer Center has held an American Cancer Society Institutional grant for many years.

***Biostatistics Core:*** Both Drs. Vogel and Luo are members of the Biostatistics Core, a shared resource of the Masonic Cancer Center with the primary objective to provide centralized biostatistics services, collaborative research, and data management support for the research projects. The shared resource serves as the focal point from which investigators and their team members and/or associates at the Masonic Cancer Center can draw biostatistics expertise for the design, data management, and analysis of their research projects.

The aims of the Biostatistics and Bioinformatics shared resource are to:

Provide biostatistics expertise in study design, including endpoint definition, sample size estimation and power calculation, randomization procedures, data collection from design, plans for report generation, interim reviews, and final analysis.

Provide biostatistics analyses and support for all cancer research projects using contemporary statistical and computing methodologies; and

Provide biomedical informatics and data management support for the development and management of all research projects, as well as projects/Core specific databases by all investigators and their team and/or associates at the Masonic Cancer Center.

Software resources for biostatistics at the Masonic Cancer Center include but are not limited to Microsoft Windows XP, Microsoft Access, Excel, PowerPoint, Word 2003, Macromedia DreamWeaver 8.0; Adobe Acrobat ; MacAfee Virus protection ; PASS, Power, CTDS, and NQuery Advisor; SAS 9.1; SPLUS 8.0; Computers are linked via Ethernet to the University system. All databases are backed-up nightly. The Biostatistics Core is supported by the Academic Health Center Information Services along with the Medical School. When appropriate we collaborate with other University of Minnesota resources including the Minnesota Supercomputing Institute (MSI) and the Office of Information Technology (OIT). We utilize a mix of operating environments depending on the application requirement, including Linux, Solaris, and Windows.

**HealthPartners and HealthPartners Institute**

**HealthPartners** is the largest consumer-governed nonprofit health care organization in the country, providing care, coverage, research, and education to improve health and well-being in partnership with its members, patients and community. Included under HealthPartners’ umbrella are Regions Hospital (a tertiary-care hospital in St. Paul, MN), Park Nicollet HealthPartners Care Group, HealthPartners Center for Memory & Aging, Park Nicollet Methodist Hospital (a 426-bed facility with more than 960 physicians in St. Louis Park, MN) and HealthPartners Institute. HealthPartners has formal relationships with hospitals and clinics throughout Minnesota and western Wisconsin, including Westfields Hospital (New Richmond, WI), Lakeview Hospital (Stillwater, MN), Hudson Hospitals and Clinics (Hudson, WI), Amery Hospital and Clinic (Amery, WI), St Francis Regional Medical Center (Shakopee, MN) and Physicians Neck and Back Clinic (locations in Roseville, Woodbury, Coon Rapids, Edina, Burnsville, and Maple Grove, MN).

Founded in 1957, the HealthPartners family of care serves more than 1.5 million medical and dental health plan members and more than 1.2 million patients. In 2013, HealthPartners and Park Nicollet Health Services combined under the name HealthPartners and a single consumer-governed board of directors. The new organization includes a multispecialty group practice of more than 1,700 physicians; 7 hospitals; 47 primary care clinics; 22 urgent care locations; 22 dental clinics; and numerous specialty practices in Minnesota and western Wisconsin. HealthPartners is the top-ranked commercial health plan in Minnesota and is ranked among the top 30 plans in the nation, according to the National Committee for Quality Assurance’s Health Insurance Plan Rankings 2013-2014. For more information, visit [www.healthpartners.com](http://www.healthpartners.com).

**HealthPartners Institute Services and Infrastructure:**

**HealthPartners Institute** (the Institute) is a 501c(3) nonprofit organization dedicated to conducting high quality, public-domain health research, often in collaboration with other academic and research organizations throughout the world. On January 1, 2016, HealthPartners Institute for Education and Research and Park Nicollet Institute combined to form one of the largest medical research and education centers in the region. The combined HealthPartners Institute employs 33 career research investigators and more than 400 clinician researchers and encompasses vast and varied areas of research including neurosciences, critical care, dental and oral health, maternal and child health, chronic disease, cancer, clinical research, health economics, mental health, Struthers Parkinson’s Center and Park Nicollet International Diabetes Center. In addition, the Institute participates in nine national research networks including the Health Care Systems Research Network, the Vaccine Safety Datalink, the Cancer Research Network and the Mental Health Research Network. For more information, visit [www.healthpartnersinstitute.org](http://www.healthpartnersinstitute.org).

A large **in-house professional support team** helps investigators with proposal development, research study coordination, accessing and processing data and a range of other needs. The professional staff includes full-time study coordinators, phone coaches, research technicians and administrative staff. An onsite Survey Research Center conducts surveys and chart review. A research librarian conducts literature searches. Office resources include more than 300 workstation computers networked with the HealthPartners- and Institute-owned computer systems that reside with HealthPartners-owned systems at a secure offsite computing facility.

The Institute administers both **external and internal grants for investigators**. A number of different government agencies, foundations and industries funds external grants, while the Institute itself awards internal grants. A small number of internal grants of up to $60,000 and up to two year project periods are awarded annually to HealthPartners or Park Nicollet employees for purposes of discovery, or research program development. Proposals are reviewed internally for science and merit and receive special consideration if they address key organizational priorities.

The Institute has an **accounting and finance** unit distinct from HealthPartners that is an independent fiscal agent for all grants and contracts. In 2016, the Institute had a budget of $37.5 million from grants and contracts from various government agencies (NIH, CDC, AHRQ), foundations and corporations.

The Institute has a team of research programmers and application developers in our **Research Informatics and Information Systems (RIIS)** department. Capabilities of this team include tracking database development, web development, SAS and SQL query and integration of elements in the electronic medical record. The team consists of four divisions: Application Development, Data Systems & Architecture, Virtual Data Warehouse (VDW) Architecture and Operations, and Research Informatics.

**Application Development:** This team focuses on Web-based applications, backend Web services, client server applications and the development and maintenance of the Institute and study-related Web sites. Its members have extensive expertise in backend database and agile software development.

**Data Systems and Architecture:** This area develops the Epic electronic medical record system, internal data structures and the Institute Data Mart. The Institute Data Mart is a long-term project to increase productivity by allowing easier, faster access to data pertinent to Institute research. The Data Systems and Architecture Team is central to our data-coordination efforts with single-site and multisite studies.

**VDW Architecture and Operations:** The VDW is a long-term effort to facilitate collaboration with other research organizations by allowing the pooling and manipulation of common data elements. This team consists of 2.25 dedicated programmer analysts, with assistance from the Research Informatics Team.

**Research Informatics:** This team works with researchers to identify and analyze information to answer research questions. Members help with proposal development and use tools and technologies to extract and combine data from different data sources to create a comprehensive record for research. Managers of this team ensure adequate staffing so that a team is convened at the start of a project.

The Institute has implemented a comprehensive internal **Regulatory Process** to ensure scientific merit and research subject safety. The Institute is one component of a larger organization defined as an affiliated covered entity (ACE) under HIPAA regulations. The Institute’s link with the Park Nicollet HealthPartners Care Group and Regions Hospital enables researchers to explore the feasibility of protocol design and identify participants for study proposals from member and patient databases. In compliance with HIPAA regulations, the Institute established a firewall between the overall data warehouse, which includes medical records and administrative data, and all research staff. As a result, only Institute programmers have direct access to protected health information as defined by HIPAA. They also have permission to release certain information to researchers in preparation for research and, once projects have received the required approvals (scientific and Institutional Review Board [IRB]), direct access to use of medical records data and other administrative data.

The Institute’s **IRB** meets twice monthly to review pending proposals. The IRB is accredited by the Association for the Accreditation of Human Research Protection Programs. The Institute also has a **Research Review Committee.** This committee evaluates the scientific importance, feasibility and likelihood of the proposed project to 1) contribute new, generalizable knowledge to the field and 2) improve the health and well-being, experience, or affordability of care for our patients, members, or the community. All applications to conduct research that involve HealthPartners patients or members, including internal grant applications, must be reviewed and approved by the research review committee.

The HealthPartners 8170 campus features multiple **seminar and conference rooms** that allow for video and audio conferencing. Conference rooms have an overhead projection system to display computer images, installed PC and also a DVD player & VCR combo to view a DVD/video through the projection system, a Cisco TelePresence System, 70’ high definition flat screen television, maneuverable mounted camera and microphones that have muting capability with indicator lights. These rooms also include easel/flipchart, e-net (LAN) computer connection for internet/server access, a digital phone to reach meeting attendees, and an analog phone line with dial out capabilities for conference calls. There are multiple training rooms for Information Systems, Member Services and Human resources provided with equipment including: PC’s, a portable projector, a manual pull down screen, easel/flipchart, TV/VCR, E-net (LAN) connection for internet/server access, and a whiteboard.

The Institute’s **Research Methodology Group (RMG)** leads the methodological and statistical activities associated with internally and externally funded projects. RMG consists of four PhD-level and two master-level methodologists. The group expertise includes epidemiologic methods, comparative effectiveness research, causal inference, biostatistics, and demography. RMG facilitates all steps of research, from grant writing, planning and execution, to analysis and writing for publications.

The Institute’s **Research Methodology Group (RMG)** leads the methodological and statistical activities associated with internally and externally funded projects. RMG consists of four PhD-level and two master-level methodologists. The group expertise includes epidemiologic methods, comparative effectiveness research, causal inference, biostatistics, and demography. RMG facilitates all steps of research, from grant writing, planning and execution, to analysis and writing for publications.

The Institute features an onsite Center for Evaluation and **Survey Research Center (CESR)** dedicated to gathering high-quality self-reported data. All data is collected in a manner designed to minimize total survey error, including error due to nonresponse and measurement. Surveys are conducted using multiple modes, including mail, telephone, Web and in-person interviews. Mixed-mode survey methodologies are also commonly employed. The CESR is traditionally involved in a project during proposal development, data collection, entry, cleaning, delivery, storage, and response tabulation. Specifically, the CESR offers these services and technologies:

- Survey research design
- Methodological consultation on mode, contact protocol and use of incentives
- Questionnaire development (e.g., question order and wording, response categories)
- Pretesting (e.g., sample, questionnaire, population, process, cover letters, response rate)
- Data collection (e.g., mail, telephone, in-person, Web mixed-mode)
- Editing of data
- Coding open-ended questions
- Scanning
- Clinical trial screening

The CESR is managed by a survey methodologist and health services researcher, Jeanette Ziegenfuss, PhD, who has 12 plus years of experience in survey research in the health field. She is an active member of the American Association of Public Opinion Research and publishes articles on methods to reduce survey error.

The CESR’s onsite phone center conducts phone interviews as a complete survey or as follow up to a mailed survey. The CESR conducts calls from 9 am to 9 pm weekdays and during the day on Saturdays. For multisite data collection, times are altered to align with different time zones. The CESR has 10 professional telephone interviewers and can conduct interviews in English, Spanish, Hmong and Somali. Across modes all data is tracked in project-specific REDCap databases. Call management is orchestrated across all projects through a proprietary call management system that wraps around REDCap. In addition, the equipment and software can create and capture survey data using scanable forms through Teleform Elite software. Project sizes vary from small studies with 1-2 subjects per month to large, complex, multistate projects with thousands of participants.

**Research Electronic Data Capture (REDCap)**: In 2011, the Institute became a REDCap consortium partner. REDCap is a secure Web application for building and managing online surveys and databases using: 1) the online method from a Web browser using the Online Designer; and/or 2) the offline method by constructing a data dictionary template file in Microsoft Excel that can be uploaded into REDCap. Both surveys and databases (or a combination) can be built using these methods. REDCap provides audit trails for tracking data manipulation and user activity and automated export procedures for data downloads to Excel, PDF, and common statistical packages (SPSS, SAS, Stata, R). Also included are a built-in project calendar, scheduling module, reporting tools and advanced features such as branching logic, file uploading and calculated fields.

**Data System Capabilities:** The Institute’s networked workstation computers communicate with the larger HealthPartners corporate network. Our systems for storing and backing up data reside both at the HealthPartners corporate headquarters and in a secure offsite facility. Data are backed up daily, weekly and monthly. Backups are saved for 7 years. The Institute’s computer and data needs are supported by the larger organization’s Information Systems & Technology Department (IS&T). IS&T maintains all HealthPartners computer hardware, software and data, including EMR and administrative data.

**Membership System:** The membership system is a corporate database of people covered by HealthPartners health insurance from 1990 to today. The membership system provides comprehensive member data collected by the health plan for administrative and financial purposes. Membership data is collected through enrollment forms, individually, by government programs or from an employer group. Sales/Marketing gathers employer group information with input from Actuarial/Underwriting. Membership Accounting enters the information into the system for use by all enterprise applications. In addition, if a patient arrives at a clinic and they are not yet enrolled, a mini-registration process allows clinic staff to register the patient, issue a chart number and proceed with treatment. The major business functions of the membership application are: 1) maintaining employer group information, 2) maintaining member information, 3) regulatory processing, and 4) membership reporting. For research, this database identifies people with HP insurance and includes address and demographic information and information allowing linkage with the EMR and other health data.

**Electronic Medical Record (EPIC/Clarity):** All medical care provided by HealthPartners-owned facilities (including clinics and hospitals) is captured and maintained in an EMR. Epic supports clinical care and tracks billable treatments. For research and other reporting purposes, a subset of this data is extracted nightly into an Epic database called Clarity. Clarity includes demographic, encounter, admission/discharge/transfer, order, medication, chemotherapy/infusion, laboratory, radiology, pathology, vital sign, diagnosis, procedure, immunization, patient-reported outcomes (e.g., PHQ-9), physician notes, dates of service, provider, facility, billing, scheduling, flowsheet, online access and social history data. Epic data elements that do not flow to Clarity can be obtained from a production server, Chronicles, which supports the EMR. Lab results go back to 1990 and encounter data goes as far back as 1997. Computerized physician order entry started in 2005. Data originating from Park Nicollet after July 2011 are available in Epic. Prior to that time, data are available in the legacy Park Nicollet electronic medical record.

**Clinical Data Warehouse (CDW):** The CDW is a unified warehouse of clinical information sourced from both the HealthPartners and Park Nicollet EMR. Standardized field names and classification schemes are used to perform advanced analytics that maximize operational efficiency.

**Administrative Data Warehouse (ADW):** The ADW is a database of claims processed by the HealthPartners health plan. These data include demographic, enrollment, medical claims, pharmacy claims, dental claims, diagnosis, procedure, dates of service, provider, facility, laboratory tests performed, referrals to other specialists and financial and other billing codes. Medical and pharmacy claims data is available back to 1990.

**Research Data Mart (RDM):** RDM is a subset of the ADW that allows efficient retrieval for standard types of research questions. The database is optimized for questions related to identification of patients with particular conditions based on codes (e.g., ICD codes, pharmacy). This dataset is a readily accessed archive of research data from the ADW back to the 1990s.

**Virtual Data Warehouse (VDW):** The VDW facilitates collaboration with other research organizations by allowing pooling and manipulation of common data elements. The VDW is “virtual” in the sense that the research data remain at each Health Care Systems Research Network (HCSRN) site; the VDW is a local database designed in collaboration with other HCSRN sites and built to a common set of standardized file definitions. Content areas and data elements represent commonly required elements for research studies. Primary content areas include enrollment, demographics, pharmacy, utilization and cancer (tumor registry). Other content areas are census, vital signs, laboratory values and death. For each content area, data specifications define the common format for each element, such as variable name, variable label, extended definition, code values and value labels. Local site programmers have mapped and transformed data elements from their local data systems into the standardized set of variable definitions, names and codes. The VDW facilitates multisite research in the HCSRN because much of the preparatory work for pooling existing data at multiple sites has been done.

# References

1. American Cancer Society. Cancer Facts and Figures 2016. In. Atlanta: American Cancer Society; 2016.

2. Gilchrest BA, Eller MS, Geller AC, Yaar M. The pathogenesis of melanoma induced by ultraviolet radiation. *The New England journal of medicine.* 1999;340(17):1341-1348.

3. Narayanan DL, Saladi RN, Fox JL. Ultraviolet radiation and skin cancer. *International journal of dermatology.* 2010;49(9):978-986.

4. Bradford PT, Freedman DM, Goldstein AM, Tucker MA. Increased risk of second primary cancers after a diagnosis of melanoma. *Arch Dermatol.* 2010;146(3):265-272.

5. Cruse CW, Wells KE, Schroer KR, Reintgen DS. Etiology and prognosis of local recurrence in malignant melanoma of the skin. *Ann Plast Surg.* 1992;28(1):26-28.

6. Soong SJ, Harrison RA, McCarthy WH, Urist MM, Balch CM. Factors affecting survival following local, regional, or distant recurrence from localized melanoma. *J Surg Oncol.* 1998;67(4):228-233.

7. Kricker A, Armstrong BK, Goumas C, et al. Ambient UV, personal sun exposure and risk of multiple primary melanomas. *Cancer causes & control : CCC.* 2007;18(3):295-304.

8. Vogel RI, Strayer LG, Engelman L, et al. Sun Exposure and Protection Behaviors among Long-term Melanoma Survivors and Population Controls. *Cancer Epidemiol Biomarkers Prev.* 2017.

9. Ferrucci LM, Vogel RI, Cartmel B, Lazovich D, Mayne ST. Indoor tanning in businesses and homes and risk of melanoma and nonmelanoma skin cancer in 2 US case-control studies. *J Am Acad Dermatol.* 2014;71(5):882-887.

10. Lazovich D, Choi K, Vogel RI. Time to get serious about skin cancer prevention. *Cancer Epidemiol Biomarkers Prev.* 2012;21(11):1893-1901.

11. Lazovich D, Vogel RI, Berwick M, Weinstock MA, Anderson KE, Warshaw EM. Indoor tanning and risk of melanoma: a case-control study in a highly exposed population. *Cancer Epidemiol Biomarkers Prev.* 2010;19(6):1557-1568.

12. Lazovich D, Vogel RI, Berwick M, Weinstock MA, Warshaw EM, Anderson KE. Melanoma risk in relation to use of sunscreen or other sun protection methods. *Cancer Epidemiol Biomarkers Prev.* 2011;20(12):2583-2593.

13. Torres SM, Luo L, Lilyquist J, et al. DNA repair variants, indoor tanning, and risk of melanoma. *Pigment Cell Melanoma Res.* 2013;26(5):677-684.

14. Vogel RI, Ahmed RL, Nelson HH, Berwick M, Weinstock MA, Lazovich D. Exposure to indoor tanning without burning and melanoma risk by sunburn history. *Journal of the National Cancer Institute.* 2014;106(6):dju112.

15. Lazovich D, Isaksson Vogel R, Weinstock MA, Nelson HH, Ahmed RL, Berwick M. Association Between Indoor Tanning and Melanoma in Younger Men and Women. *JAMA dermatology.* 2016;152(3):268-275.

16. Vogel RI, Strayer LG, Ahmed RL, Blaes A, Lazovich D. A Qualitative Study of Quality of Life Concerns following a Melanoma Diagnosis. *Journal of skin cancer.* 2017;2017:2041872.

17. Vogel RI, Strayer LG, Engelman L, et al. Comparison of quality of life among long-term melanoma survivors and non-melanoma controls: a cross-sectional study. *Quality of life research : an international journal of quality of life aspects of treatment, care and rehabilitation.* 2017;26(7):1761-1766.

18. Vogel RI, Strayer LG, Engelman L, et al. Sun Exposure and Protection Behaviors among Long-term Melanoma Survivors and Population Controls. *Cancer Epidemiol Biomarkers Prev.* 2017;26(4):607-613.

19. Glanz K, Schoenfeld ER, Steffen A. A randomized trial of tailored skin cancer prevention messages for adults: Project SCAPE. *Am J Public Health.* 2010;100(4):735-741.

20. Glanz K, Volpicelli K, Jepson C, Ming ME, Schuchter LM, Armstrong K. Effects of tailored risk communications for skin cancer prevention and detection: the PennSCAPE randomized trial. *Cancer Epidemiol Biomarkers Prev.* 2015;24(2):415-421.

21. Heckman CJ, Darlow SD, Ritterband LM, Handorf EA, Manne SL. Efficacy of an Intervention to Alter Skin Cancer Risk Behaviors in Young Adults. *Am J Prev Med.* 2016;51(1):1-11.

22. Fogg B. A behavior model for persuasive design. Proceedings of the 4th International Conference on Persuasive Technology; 2009; Claremont, California, USA.

23. Holman DM, Berkowitz Z, Guy GP, Jr., Hartman AM, Perna FM. The association between demographic and behavioral characteristics and sunburn among U.S. adults - National Health Interview Survey, 2010. *Prev Med.* 2014;63:6-12.

24. Lloyd-Jones DM, Hong Y, Labarthe D, et al. Defining and setting national goals for cardiovascular health promotion and disease reduction: the American Heart Association's strategic Impact Goal through 2020 and beyond. *Circulation.* 2010;121(4):586-613.

25. Zigmond AS, Snaith RP. The hospital anxiety and depression scale. *Acta psychiatrica Scandinavica.* 1983;67(6):361-370.

26. Fogg BJ. A Behavior Model for Persuasive Design. In: University S, ed. *Persuasive*. Clairemont, CA2009.

27. Banerjee S, Hoch EG, Kaplan PD, Dumont EL. A comparative study of wearable ultraviolet radiometers. Paper presented at: 2017 IEEE Life Sciences Conference (LSC)2017.

28. Stump TK, Aspinwall LG, Gray EL, et al. Daily Minutes of Unprotected Sun Exposure (MUSE) Inventory: Measure description and comparisons to UVR sensor and sun protection survey data. *Prev Med Rep.* 2018;11:305-311.

29. Day AK, Wilson CJ, Hutchinson AD, Roberts RM. The role of skin cancer knowledge in sun-related behaviours: a systematic review. *J Health Psychol.* 2014;19(9):1143-1162.

30. Gilchrest BA. *Skin and aging process.* CRC Press; 1984.

31. Parkin DM, Mesher D, Sasieni P. 13. Cancers attributable to solar (ultraviolet) radiation exposure in the UK in 2010. *British journal of cancer.* 2011;105 Suppl 2:S66-69.

32. Godar DE, Urbach F, Gasparro FP, van der Leun JC. UV doses of young adults. *Photochemistry and photobiology.* 2003;77(4):453-457.

33. Harris PA, Taylor R, Thielke R, Payne J, Gonzalez N, Conde JG. Research electronic data capture (REDCap) - A metadata-driven methology and workflow process for providing translational research informatics support. *J Biomed Inform.* 2009;42(2):377-381.

34. van der Swaluw K, Lambooij MS, Mathijssen JJP, Zeelenberg M, Polder JJ, Prast HM. Emotional responses to behavioral economic incentives for health behavior change. *Psychol Health Med.* 2018:1-10.

35. Bjelland I, Dahl AA, Haug TT, Neckelmann D. The validity of the Hospital Anxiety and Depression Scale. An updated literature review. *J Psychosom Res.* 2002;52(2):69-77.

36. Schafer JL. *Analysis of Incomplete Multivariate Data.* New York: Chapman and Hall; 1997.

37. Little RJA, Rubin DB. *Statistical Analysis with Missing Data.* New York: John Wiley & Sons, Inc.; 1987.

38. Molenberghs G, Kenward MG. *Missing Data in Clinical Studies.* New York: John Wiley & Sons, Ltd.; 2007.

**Appendix: Summary of Protocol Changes and Rationale – 01/27/2020**

Major changes:

1. The original version of this protocol was funded by the American Cancer Society. We also received funding for usability and a pilot study version of this proposed work from the Melanoma Research Alliance (protocol STUDY00002107/2018NTLS002). We negotiated to combine the funding to support one larger study to increase our power for experience of sunburn during the intervention period, a key secondary outcome. Therefore, we have increased the sample size here from 248 to 368 (184 each wave/summer) and we will formally close the previous pilot study with the CPRC, IRB and CT.gov and add the Melanoma Research Alliance as a funding source for this protocol. The PI discussed this plan of action with Felicia Mroczkowski on 1/24/2020 who agreed this would likely be the best path from the IRB perspective.
2. After our usability testing we have decided to switch to a different wearable device than originally proposed. This device, Shade, was developed in collaboration with the NIH NCI and has been shown to be significantly more accurate than our previously proposed device. One disadvantage of this device is that it does not objectively measure physical activity as the previous device did and therefore those pieces were removed from the protocol. We will rely on self-report measures of physical activity to test our secondary hypotheses related to physical activity. Additionally, this device is significantly more expensive than the previously planned device and therefore participants will be asked to return the device at the end of the intervention period and be provided gift cards throughout the study as incentive instead. They will be provided up to $120 in gift cards if they complete all study procedures ($20 baseline survey, $10 week 4 survey, $10 week 8 survey, $60 return device and complete week 12 survey, $20 complete week 64 survey).
3. As this is a minimal risk study, we have removed the during-study monitoring of anxiety and depression. We have found in another study of cancer survivors that this monitoring and sending of letters often confuses and distresses participants. We will plan, however, to examine whether rates of anxiety and/or depression rise during the intervention period (week 12 vs. baseline) following Wave 1. While we do not expect that to be the case, should it happen we will proactively monitor in Wave 2.

Minor changes:

1. In addition to providing information about potential barriers to sun protection to those in the intervention via the website, we will also provide this information in a paper brochure that has been reviewed with melanoma survivors as part of usability testing.
2. We finalized our survey measures. They are similar in topic to what was originally proposed but careful thought was put into reducing the number of items while maintaining focus on our primary and secondary objectives. Table 2 had been updated accordingly.
3. Initial recruitment contact from the University of Minnesota will be via mail as opposed to phone. This change was made to ensure we have proper mailing addresses prior to sending the device and requires participants to take one additional step (return consent/HIPAA forms by mail) which we hope will translate to an increased chance they will return the device at the end of the study.
4. We have clarified that all recruitment will be done through HealthPartners; they will be the single site of recruitment. We also clarified that HealthPartners will be requesting a waiver of consent to identify potentially eligible participants and a waiver of documentation of written consent to provide contact information to the University of Minnesota for those who verbally agree to participate.
5. We have provided all final participant communications.
